# CircHERC1 promotes non-small cell lung cancer cell progression by sequestering FOXO1 in the cytoplasm and regulating the miR-142-3p-HMGB1 axis

**DOI:** 10.1186/s12943-023-01888-7

**Published:** 2023-11-06

**Authors:** Yumeng Cui, Xiaojie Wu, Jie Jin, Weiling Man, Jie Li, Xiang Li, Yanghua Li, He Yao, Rongbin Zhong, Shiyun Chen, Jiahui Wu, Tianhao Zhu, Yanli Lin, Junjie Xu, Youliang Wang

**Affiliations:** 1 Beijing Institute of Biotechnology, Beijing, 100071 China; 2 Department of Thoracic Surgery, The First Medical Center of Chinese PLA General Hospital, Beijing, 100850 China

**Keywords:** circRNA, Non-small cell Lung cancer, miRNA, Nuclear and cytoplasmic localization

## Abstract

**Background:**

Noncoding RNAs such as circular RNAs (circRNAs) are abundant in the human body and influence the occurrence and development of various diseases. Non-small cell lung cancer (NSCLC) is one of the most common malignant cancers. Information on the functions and mechanism of circRNAs in lung cancer is limited; thus, the topic needs more exploration. The purpose of this study was to identify aberrantly expressed circRNAs in lung cancer, unravel their roles in NSCLC progression, and provide new targets for lung cancer diagnosis and therapy.

**Methods:**

High-throughput sequencing was used to analyze differential circRNA expression in patients with lung cancer. qRT‒PCR was used to determine the level of circHERC1 in lung cancer tissues and plasma samples. Gain- and loss-of-function experiments were implemented to observe the impacts of circHERC1 on the growth, invasion, and metastasis of lung cancer cells in vitro and in vivo. Mechanistically, dual luciferase reporter assays, fluorescence in situ hybridization (FISH), RNA immunoprecipitation (RIP) and RNA pull-down experiments were performed to confirm the underlying mechanisms of circHERC1. Nucleocytoplasmic localization of FOXO1 was determined by nucleocytoplasmic isolation and immunofluorescence. The interaction of circHERC1 with FOXO1 was verified by RNA pull-down, RNA immunoprecipitation (RIP) and western blot assays. The proliferation and migration of circHERC1 in vivo were verified by subcutaneous and tail vein injection in nude mice.

**Results:**

CircHERC1 was significantly upregulated in lung cancer tissues and cells, ectopic expression of circHERC1 strikingly facilitated the proliferation, invasion and metastasis, and inhibited the apoptosis of lung cancer cells in vitro and in vivo. However, knockdown of circHERC1 exerted the opposite effects. CircHERC1 was mainly distributed in the cytoplasm. Further mechanistic research indicated that circHERC1 acted as a competing endogenous RNA of miR-142-3p to relieve the repressive effect of miR-142-3p on its target HMGB1, activating the MAPK/ERK and NF-κB pathways and promoting cell migration and invasion. More importantly, we found that circHERC1 could bind FOXO1 and sequester it in the cytoplasm, adjusting the feedback AKT pathway. The accumulation of FOXO1 in the cytosol and nuclear exclusion promoted cell proliferation and inhibited apoptosis. CircHERC1 is a new circRNA that promotes tumor function in NSCLC and may serve as a potential prognostic biomarker and therapeutic target for NSCLC.

**Conclusions:**

CircHERC1 is a new circRNA that promotes tumor function in NSCLC and may serve as a potential diagnosis biomarker and therapeutic target for NSCLC. Our findings indicate that circHERC1 facilitates the invasion and metastasis of NSCLC cells by regulating the miR-142-3p/HMGB1 axis and activating the MAPK/ERK and NF-κB pathways. In addition, circHERC1 can promote cell proliferation and inhibit apoptosis by sequestering FOXO1 in the cytoplasm to regulate AKT activity and BIM transcription.

**Supplementary Information:**

The online version contains supplementary material available at 10.1186/s12943-023-01888-7.

## Introduction

According to the latest statistics, lung cancer, of which 85% are non-small cell lung cancers(NSCLC), is the most commonly diagnosed cancer and the leading cause of cancer death in both men and women, the incidence and mortality of lung cancer have increased due to environmental pollution and an increasing number of smokers in recent years [1].The understanding of genetic alterations that drive NSCLC is evolving. New strategies in molecular diagnostics and targeted therapies in NSCLC to detect and treat them are being explored. These therapeutic molecules serve as important sources for new drugs that interfere with cellular proliferation, apoptosis, metastasis, and angiogenesis by regulating signaling pathways. Despite extensive research and progress, therapies for lung cancer remain inadequate and further improvements are needed [2]. Profiling the molecular aberrations in plasma of lung cancer patients could provide important biologic insights into lung tumorigenesis, and potential biomarkers and therapeutic targets for the disease.

Noncoding RNAs (ncRNAs) are a large and diverse class of transcribed RNA molecules and participate in many biological processes [3]. CircRNAs are a class of noncoding RNA molecules that do not have a 5’ terminal cap and a 3’ terminal poly(A) tail and form a ring structure by covalent bonds and are present objectively in organisms [4, 5]. The unique circular structure of circRNAs confers inherent resistance to degradation by exonuclease and makes circRNAs more stable than their linear parental genes [6]. CircRNAs are abundant in the human body and have multiple biological functions [7–9]. The landscape and action of circRNAs have been widely reported in various types of cancer [10], suggesting that circRNAs may be potential biomarkers or therapeutic targets [5, 11].The function of circRNAs is usually associated with their source and location in the cells. Most circRNAs are derived from the exon(s) of genes and are thus called exonic circRNAs (ecRNAs), which are located in the cytoplasm [12]. There is an interactive relationship between microRNAs (miRNAs) and circRNAs, referred to as the “miRNA sponge” effect [13–15]. During this process, circRNAs trap miRNAs on specific binding sites, thus preventing miRNAs from interfering with mRNA expression. This process can mediate cancer progression [14, 16, 17]. In addition, circRNAs can interact with RNA binding proteins and participate in the development of different cancers [8, 13, 18–20]. Studies have shown that some circRNAs can affect the distribution of nuclear matter by causing nuclear/cytoplasmic retention. In addition, circRNAs can transport proteins to nucleoli, mitochondria and the cell membrane [21]. Research on the interaction between circRNAs and protein is still in the initial stage. The circRNA‒protein interaction changes the protein so that it can regulate vital activities, which has inspired us to design new clinical strategies based on interference with or utilization of this interaction [22, 23].

With the deepening of research, researchers have revealed that circRNAs can affect the incidence and development of lung cancer through a variety of mechanisms [5, 24]. In this study, we used RNA sequencing (RNA-seq) to analyze circRNA profile of NSCLC patients’ plasma and identified the upregulated circHERC1, which is derived from the exons of the HERC1 gene. CircHERC1 was significantly upregulated in lung cancer tissues and cells, ectopic expression of circHERC1 strikingly facilitated the invasion, and metastasis of lung cancer cells in vitro and in vivo. Further mechanistic research indicated that circHERC1 facilitated the invasion and metastasis of NSCLC cells by regulating the miR-142-3p/HMGB1 axis and activating the MAPK/ERK and NF-κB pathways. More importantly, we found that circHERC1 could bind to FOXO1 and sequester it in the cytoplasm, thereby promoting the proliferation of lung cancer cells. This is the first report on the expression, role and regulatory mechanism of hsa_circHERC1 in NSCLC. The findings of this study further expanded the research on the significance of circRNAs in NSCLC progression and may bring new insights for the exploitation of novel biomarkers and therapeutics for NSCLC.

## Materials and methods

### Clinical specimens

16 tumors and matched adjacent samples of NSCLC patients were collected from Chinese PLA General Hospital. Blood samples were collected from patients with lung cancer and benign nodules who were hospitalized. All patients obtained written informed consent. Detailed patient records were recorded and kept during specimen collection. According to the clinical data, patients with other cancers, patients with previous cancer history or infectious virus infection, patients with non-small cell lung adenocarcinoma with surgery, radiotherapy, chemotherapy, or drug treatment were excluded. 188 blood samples from patients with non-small cell lung cancer and 53 blood samples from patients with benign nodules were collected, and plasma was separated, and stored in an RNase free tube for cryopreservation. This study was authorized by the Ethics Committee of Chinese PLA General Hospital and the Ethics Committee of Beijing Institute of Biotechnology.

### Cell culture

Immortal human bronchial epithelial cells (BEAS-2B), and human lung cancer cell lines including A549, NCI-H460 and NCI-H1299 were purchased from the American Type Culture Collection (ATCC, USA). Lung cancer cells including NCI-H3255, HCC-1588 and Calu-3 cells, were purchased from Shanghai Cell Bank. All cells were maintained at 37 °C under 5% CO_2_ and at least 95% humidity. BEAS-2B, A549, NCI-H460, NCI-H1299 and HCC-1588 cells were cultured in DMEM (HyClone, USA), and NCI-H3255 and Calu-3 cells were cultured in 1640 medium (Gibco, USA) supplemented with 10% fetal bovine serum (Gibco, USA).

### RNA‑seq

For circRNA sequencing, total RNA was extracted from plasma with a RNeasy® Kit (Qiagen, GER). The RNA was then treated using a Ribo-off rRNA Depletion Kit (Vazyme, China) to remove ribosomal RNA before generating the RNA-seq library. Then, an Illumina HiSeq^TM^2500 instrument (Illumina, USA) was used to perform library sequencing according to the manufacturer’s instructions. The FASTQ reads were aligned to the human reference genome (hg38/GRCh38). The counts of the remaining reads were normalized and mapped across an identified back-splice junction. The transcripts overlapping with the exons of the database annotations were screened out, and the transcripts overlapping with the exons of the spliced transcripts in the database were included in the follow-up analysis. The expression of each transcript was calculated through Cuffquant, and the transcripts with FPKM ≥ 0.5 were selected. The overall distribution of different circRNA was visually analyzed by volcanic map, and the different circRNA was screened from two aspects: the difference multiple and the corrected significant level.

### RNA preparation and qRT‒PCR

Total RNA was extracted from cultured cells with an Ambion PureLink™ Total RNA Kit (Thermo Scientific, USA) and quantified using a NanoDrop1000 spectrophotometer (NanoDrop Technologies, USA). For analysis of circRNA and mRNA, cDNA was randomly or oligo(dT) primed from 1 µg of total RNA using a High-Capacity cDNA Reverse Transcription Kit (Invitrogen, USA) following the manufacturer’s instructions. For analysis of miRNA, 1 µg of RNA was converted to cDNA using Mir-X™ miRNA First-Strand Synthesis (Takara, Japan) based on the manufacturer’s procedure. The cDNA was amplified by qPCR using Thunderbird SYBR® qPCR Mix (Toyobo, Japan) on a CFX96 Real-Time PCR System (Bio-Rad, USA). GAPDH and U6 were used as internal references and calculated using the 2^−ΔΔCt^ method.

### RNase R treatment

Total RNA was isolated from tissues and cells using TRIzol® reagent (Invitrogen, USA) according to the manufacturer’s protocol. cDNA was synthesized with random primers or miRNA‒specific stem‒loop primers using a Revert Aid First Strand cDNA Synthesis Kit (Thermo Scientific, USA). qRT‒PCR was performed on the Bio-Rad CFX96™ Real-Time PCR System (BioRad, USA). The primers for mRNA and circRNA were synthesized by BGI (BGI, China). To verify the ring structure of circHERC1, for RNase R treatment, 2 µg total RNA was digested with 3 U RNase R (Epicenter Technologies, USA) at 37 °C for 20 min and 70 °C for 5 min according to the manufacturer’s instructions. For controls, 2 µg of total RNA was mock treated under the same conditions without the enzyme. Then, the expression levels of linear mRNA and circular RNA were determined by qRT‒PCR and RT‒PCR.

### Cytoplasmic and nuclear fractions

Cytoplasmic and nuclear RNAs were purified with Cytoplasmic & Nuclear RNA Purification Kit (Norgen, Canada), converted to cDNAs and analyzed by qPCR, U6 served as the nuclear RNA marker, and GAPDH served as the cytoplasmic RNA marker. Cytoplasmic and nuclear proteins were extracted by nuclear and cytoplasmic extraction reagents according to the manufacturer’s instructions (Thermo Scientific, USA).

### Fluorescence in situ hybridization (FISH)

To investigate the intracellular distribution of circHERC1 in lung cancer, FAM-labeled circHERC1 and CY3-labeled miRNA-142-3p probes were synthesized by Suzhou GenePharma Co. Ltd (GenePharma, China). Cells were grown to 60–80% confluence and then fixed with 4% paraformaldehyde. The hybridization experiments were performed using a Fluorescence In Situ Hybridization Kit (GenePharma, China) according to the manufacturer’s protocol. The cell slices were mounted and images were acquired using a Nikon ECLIPSE confocal microscope (Nikon, Japan).

### Cell transfection and generation of stable cell lines

Plasmids, siRNAs, miRNA mimics or inhibitors were transfected into cells using Lipofectamine® 2000 Transfection Reagent (Thermo Scientific, USA) according to the manufacturer’s instructions. To construct cell lines stably overexpressing circHERC1, we transfected the control vector pLC5-circ or the circHERC1 overexpression vector, and single clones were selected for more than 4 weeks by incubation with 2 µg/ml puromycin (Invitrogen, USA). To generate stable circHERC1 knockdown cell lines, the control vector or sh-circHERC1 was transfected into NCI-H3255 cells, and single clones were selected for more than 4 weeks by incubation with 500 ng/ml hygromycin (Invitrogen, USA). Once fully infected, stable cells were cryopreserved or used for other experiments and assays.

### 5-Ethynyl-2’-deoxyuridine (EdU) assay

The cells to be detected were seeded on 4-well slides with chambers in advance and 10 µM EdU were added. The cells were incubated for 2 h and then stained with Azide Alexa Fluor 594 for 30 min and DAPI for 10 min using BeyoClick™ EdU Cell Proliferation Kit with Alexa Fluor 594 (Reyotime, China) according to the manufacturer’s protocol. The cells were visualized by fluorescence microscopy. The proliferation rate of cells was determined by calculating the ratio of EdU-positive nuclei to total nuclei.

### Cell viability assay

NCI-H3255 and A549 cells were collected and inoculated into 96-well plates at a concentration of 2000 cells per well, and 10 µL CCK-8 solution (Dojindo, Japan) was added to each well at 0 h, 24 h, 48 h, 72 h, 96 h. The timing was started after the cells were attached to the well. The cells were incubated at 37 °C for 2 h. The absorbance at 450 nm was measured with a microplate reader.

### Apoptosis detection

Cells were harvested by centrifugation at 1000 × g for 5 min, gently resuspended in PBS and counted. A total of 50,000-100,000 suspended cells were centrifuged at 1000 × g for 5 min, and the supernatant was discarded. Then, 195 µL binding solution, 5 µL Annexin V-FITC and 10 µL propyl iodide solution (Beyotime, China) were added to the cells and mixed gently. The mixtures were incubated at room temperature (20–25 °C) in darkness for 10–20 min and then subjected to flow cytometry analysis quickly.

### Transwell migration and invasion assay

To determine the migration and invasion of cells, 5 × 10^4^ cells were plated in medium without serum in the top chamber of a transwell with an 8.0 μm pore size (Corning, USA). Matrigel (BD Biosciences, USA) coating to perform the invasion assays. The bottom chamber contained standard medium with 20% FBS medium. After 16–24 h of culture, the cells were fixed with 90% ethanol and stained with 0.1% crystal violet (Solarbio Technology, China).

### Wound healing assays

A wound healing assay was performed in 6-well plates (5 × 10^5^ cells per well). When transfected cancer cells had grown to 90 to 95% confluence, wound lines were manually created by scratching the monolayer with a sterile 200 µl pipette tip. The migration of the cells was assessed after 24, 48, and 72 h. Pictures were taken using an inverted phase-contrast microscope. The distance between the parallel lines was measured using Image J software. All experiments were carried out at least three times. The wound healing rate (%) was calculated according to the formula: (the wound area at 0 h minus the wound area at 24 h) / the wound area at 0 h.

### Western blot

Total proteins were extracted with RIPA Lysis Buffer (Thermo Scientific, USA) and quantified using a Pierce BCA Protein Assay Kit (Thermo Scientific, USA). The total protein was separated by SDS‒PAGE and transferred onto PVDF membranes (Millipore, USA). After they were blocked in 5% nonfat milk for 1 h, the membranes were incubated 1 h at room temperature with the indicated primary antibodies, namely, anti-HMGB1 (Abcam, ab228624, 1:5000 dilution), anti-GAPDH (Cell Signaling,#2118,1:2000 dilution), anti-FOXO1(Cell Signaling, #2880,1:1000 dilution), anti-phospho-FOXO1 (Cell Signaling, #84192,1:1000 dilution), anti-phospho-AKT (Cell Signaling, #4060, 1:2000 dilution), anti-phospho-IKBα (Cell Signaling, #4814,1:1000 dilution), anti-phospho-p44/42 MAPK(ERK1/2) (Cell Signaling, #4370, 1:1000 dilution), anti-p44/42 MAPK(ERK1/2) (Cell Signaling, #4695, 1:1000 dilution), anti-AKT (Cell Signaling,#4691, 1:1000 dilution), anti-IKBα (Cell Signaling, #4814, 1:1000 dilution), anti-p65 (Cell Signaling, #3033, 1:1000 dilution), anti-phospho-p65 (Cell Signaling, #8042, 1:1000 dilution), anti-CDK2 (Cell Signaling, #18,048, 1:1000 dilution), anti-Lamin B1 (Cell Signaling, #17,416, 1:1000 dilution), and anti-BIM (Cell Signaling, #2933, 1:1000 dilution). Next, they were incubated with horseradish peroxidase (HRP)-conjugated secondary antibodies for 1 h at room temperature and visualized with the enhanced chemiluminescence (ECL) detection reagent (PerkinElmer, USA).

### RNA immunoprecipitation (RIP)

RIP assays were conducted using a Magna RIP RNA-Binding Protein Immunoprecipitation Kit (Millipore, USA) according to the manufacturer’s instructions. A total of 1 × 10^7^ A549 and NCI-H3255 cells were harvested and lysed in RIP lysis buffer containing protease inhibitor cocktail (Thermo, Germany) and RNA inhibitor (Beyotime, China), the supernatants were collected after centrifugation for 10 min at 13 000 rpm, and then incubated with Dynabeads protein G conjugated with anti-IgG or anti-AGO2 using a Magna RIP™ kit (Merck, USA). The immunoprecipitated RNAs were extracted for the detection of miRNA and circRNA expression by qRT‒PCR.

### RNA/protein pulldown

RNA pulldown was conducted as previously described [25]. Briefly, we designed a biotin-labeled 30 nt probe against the back-spliced junction of circHERC1 to specifically pull down circHERC1 and its intracellular RNA‒RNA or RNA‒protein complex. A biotin-labeled probe with a scrambled sequence was used as a negative control. A total of 1 × 10^7^ cells were cross-linked in ice-cold PBS buffer with 1% formaldehyde for 10 min. Upon PBS buffer removal, these cells were lysed in RNA immunoprecipitation (RIP) buffer on ice for 30 min. After sonication, the cell supernatant was harvested and divided into two equal parts for subsequent RNA pulldown after centrifugation. The biotin-labeled and control probes were incubated with the respective cell lysate for 4 h at 4 °C with gentle rotation. Identically blocked M280 Streptavidin magnetic Dynabeads (Invitrogen, USA) were added to the above lysates and further rotated for 4 h at 4 °C. After washing with RIP buffer and RIP buffer supplemented with 500 mM NaCl, the bound RNA was isolated using TRIzol® and used for RNA detection by RT-qPCR assay. The protein was extracted and analyzed by western blotting.

### Dual luciferase reporter assay

The sequence of circHERC1 (or the 3’ UTR of HMGB1) was subcloned into the psiCHECK2 vector (Promega, USA) to build the corresponding wild-type (WT) vectors. In the mutant vectors, the miR-142-3p binding sites in circHERC1 or the 3’ UTR of HMGB1 were mutated in the psiCHECK2 vectors. 293T cells were transfected with these vectors and Renilla expression plasmids per well using Lipofectamine® 2000 in 24-well plates. After 24 h of transfection, the cells were lysed with passive lysis buffer (Promega, USA), and reporter gene expression was assessed using a Dual Luciferase Reporter Assay System (Promega, USA).

### Hematoxylin and eosin (H&E) and immunohistochemical staining

Paraffin-embedded tissues were sectioned at a thickness of 5 μm, deparaffinized and rehydrated. For antigen retrieval, the slides were heated at 100 °C in 0.01 M citrate buffer, and 3% hydrogen peroxide was used to quench the peroxidase activity for 20 min. The sections were treated with normal goat serum and then incubated overnight with antibodies at 4 °C. After being rinsed with PBS, the sections were incubated with goat anti-rabbit IgG for 1 h. After hematoxylin counterstaining was completed, all sections were dehydrated and sealed.

### Tumorigenicity assays in nude mice

Ten athymic Nu/Nu mice (Beijing Vital River Laboratory Animal Technology Co., Ltd. China) per group were subcutaneously inoculated with 1 × 10^6^ NCI-H3255 cells transfected with control, sh-circHERC1, sh-HMGB1, sh-FOXO1, or circHERC1, or cotransfected with circHERC1 and sh-HMGB1; circHERC1 and sh-FOXO1 or circHERC1, sh-FOXO1 and sh-HMGB1. The mice were kept in a standard barrier environment; tumor growth was metered via a digital caliper every 7 days. The mice were observed for 27 days and then euthanized under deep anesthesia with pentobarbital (Sigma, USA) and the tumor was removed to measure the tumor weight and volume. The volume of the tumors was calculated by using the formula width×width×length×0.52. The animal protocol was designed to minimize pain or discomfort to the animals. All animal procedures were performed under the ethical guidelines of Beijing Institute of Biotechnology and according to the recommendations of the Beijing Experimental Animal Regulation Board. All animals were euthanized by barbiturate overdose (intravenous injection, 150 mg/kg pentobarbital sodium) for tissue collection.

### In vivo metastatic model

For the metastasis model, female Nu/Nu mice (6 weeks) were maintained in pathogen-free conditions. Mice were injected through the tail vein with control and circHERC1, sh-HMGB1, or sh-FOXO1 treated NCI-H3255 luciferase cells (1 × 10^6^) (n = 6/per group). The bioluminescence was monitored weekly.

### Statistical analysis

All experiments in this study were performed independently with at least three biological replicates. The data are presented as the means ± standard deviation. Statistical analyses were performed using GraphPad Prism. Differences between two groups were analyzed by independent sample t-tests, and differences among multiple groups were analyzed by one-way ANOVA. A value of P < 0.05 indicated a statistically significant difference.

## Results

### Identification and characteristics of circHERC1 in NSCLC

**Fig. 1:**
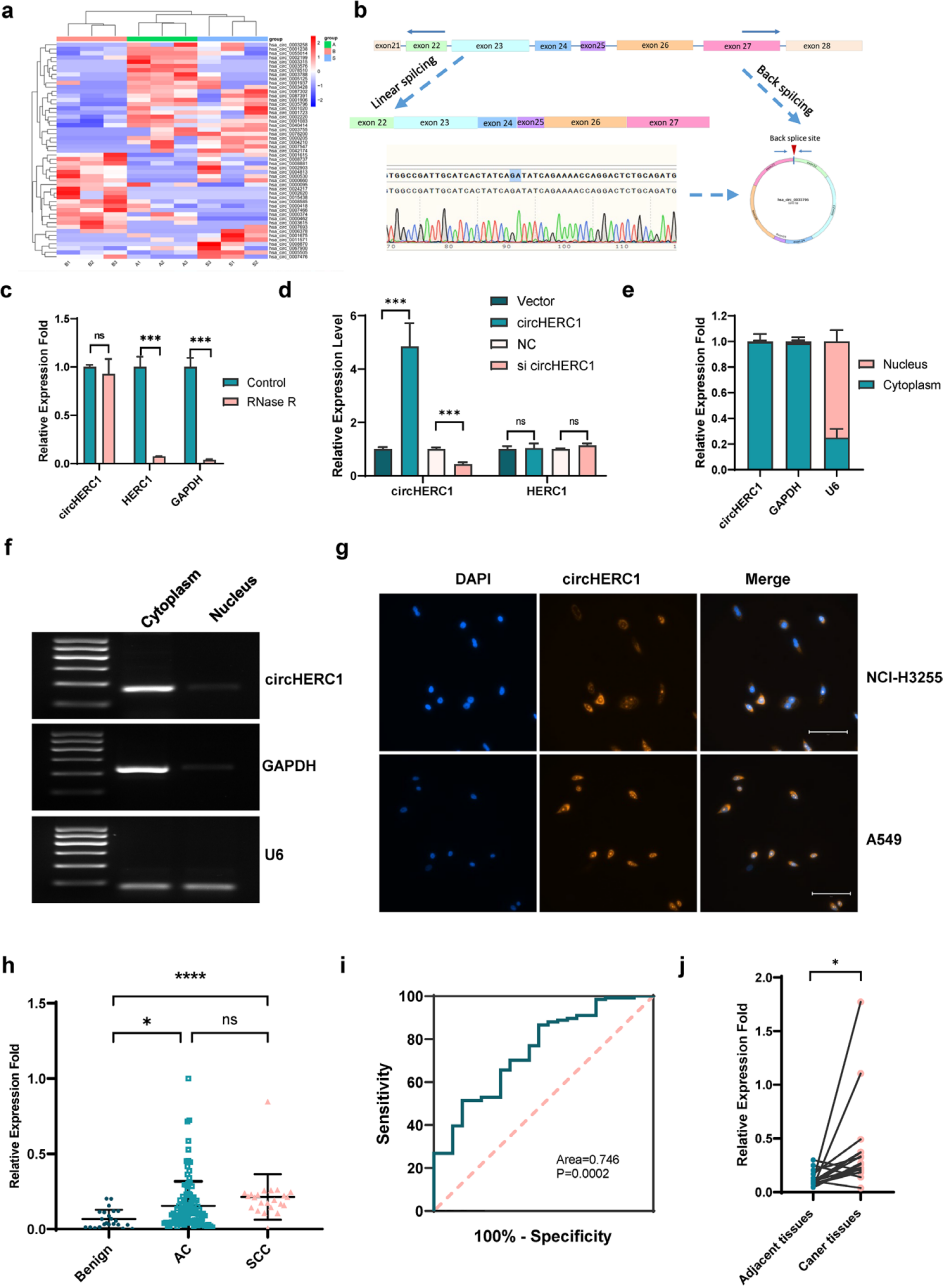
Oncogenic circRNA discovery and characterization of circHERC1 in NSCLC. (**a**) Heatmap of RNA-Seq data of differentially expressed circRNAs generated from plasma from lung cancer patients and cancer-free individuals. (**b**) Schematic illustration indicating the generation of circHERC1 from its host gene HERC1 and validation by Sanger sequencing. (**c**) qRT‒PCR assessment of the stability of circHERC1 and HERC1 under RNase R treatment in NCI-H3255 cells. ***p < 0.001. (**d**) qRT‒PCR detection of the expression of circHERC1 and its parent gene after circHERC1 overexpression or knockdown. ***p < 0.001. (**e, f**) qRT‒PCR analysis of circHERC1, GAPDH, and U6 in the cytoplasm and nucleus in NCI-H3255 cells. (**g**) FISH assay showed that circHERC1 primarily localized in the cytoplasm of NCI-H3255 and A549 cells. The scale bars are 25 μm. (**h**) qRT‒PCR was applied to analyze circHERC1 expression in plasma of LUAD patients (n = 111), LUSC patients (n = 23) and cancer-free individuals (n = 23). *p < 0.05, ****p < 0.0001. (**i**) Receiver-operating characteristic (ROC) curve analysis of circHERC1 expression in lung cancer (n = 134) and cancer-free patient (n = 23) plasma. (**j**) qRT‒PCR analysis of circHERC1 in tumor tissues and peritumoral tissues (n = 16). *p < 0.05

To investigate the role of circRNAs in NSCLC tumorigenesis, we performed plasma RNA-seq analyses of total RNA obtained from plasma samples of 3 patients with lung adenocarcinoma, 3 patients with lung squamous cell carcinoma and 3 cancer-free patients, as shown in Fig. 1a. A total of 51 distinct circRNAs were detected, of which 33 circRNAs were upregulated and 18 were downregulated in plasma from NSCLC patients compared with plasma from cancer-free patients (Supplementary Table 1). Among all of the differentially expressed circRNAs, hsa_circ_0035796 was significantly upregulated in the plasma of both patients with lung squamous cell carcinoma (LUSC) and patients with lung adenocarcinoma (LUAD) (Fig. 1a). Hsa_circ_0035796, named circHERC1, was composed of HERC1 exon 22 to exon 27 and located on the sense strand of chromosome 15 with a length of 1143 nt. The putative circHERC1 back-spliced junction was validated by PCR amplification with divergent primers from cDNA of a lung cancer cell line and confirmed by Sanger sequencing (Fig. 1b). The sequence was consistent with the circBase database annotation (http://www.circbase.org/). In NCI-H3255 and A549 cells, circHERC1 also showed resistance to RNase R (Fig. 1c and Fig. S1a) which confirmed that circHERC1 harbored a closed-loop structure [26, 27]. The expression of circHERC1, but not the parent gene, was changed (Fig. 1d). qRT‒PCR analysis of nuclear and cytoplasmic fractionation of circHERC1 and detection of agarose gel electrophoresis revealed major cytoplasmic enrichment of circHERC1 in A549 and NCI-H3255 cells (Fig. 1e-f and Fig. S1b-c). FISH examination revealed that circHERC1 was predominantly localized in the cytoplasm (Fig. 1g).

qRT‒RCR was used to verify the expression of circHERC1 in the plasma of patients. It was found that the expression of circHERC1 in plasma was higher in patients with LUSC and LUAD than in cancer-free patients (Fig. 1h). A ROC curve of the diagnostic accuracy of circRNA was drawn according to the results (Fig. 1i). The individual circRNAs exhibited AUC values of 0.746 in distinguishing malignant patients from cancer-free patients. Pairing verification between lung cancer tissues and adjacent tissues showed that the expression of circHERC1 in lung cancer tissues was higher than that in adjacent tissues (Fig. 1j). According to its occurrence and development, lung cancer can be divided into an early stage and a late stage. The expression of circHERC1 in plasma exosomes from early-stage and late-stage of lung cancer patients was significantly higher than that in plasma exosomes from cancer-free patients, but there was no significant difference between the expression of circHERC1 in early-stage lung cancer patients and that in late-stage lung cancer patients (Fig. S1d).

### CircHERC1 promotes proliferation, migration and invasion of NLCSC cells and inhibits apoptosis

**Fig. 2:**
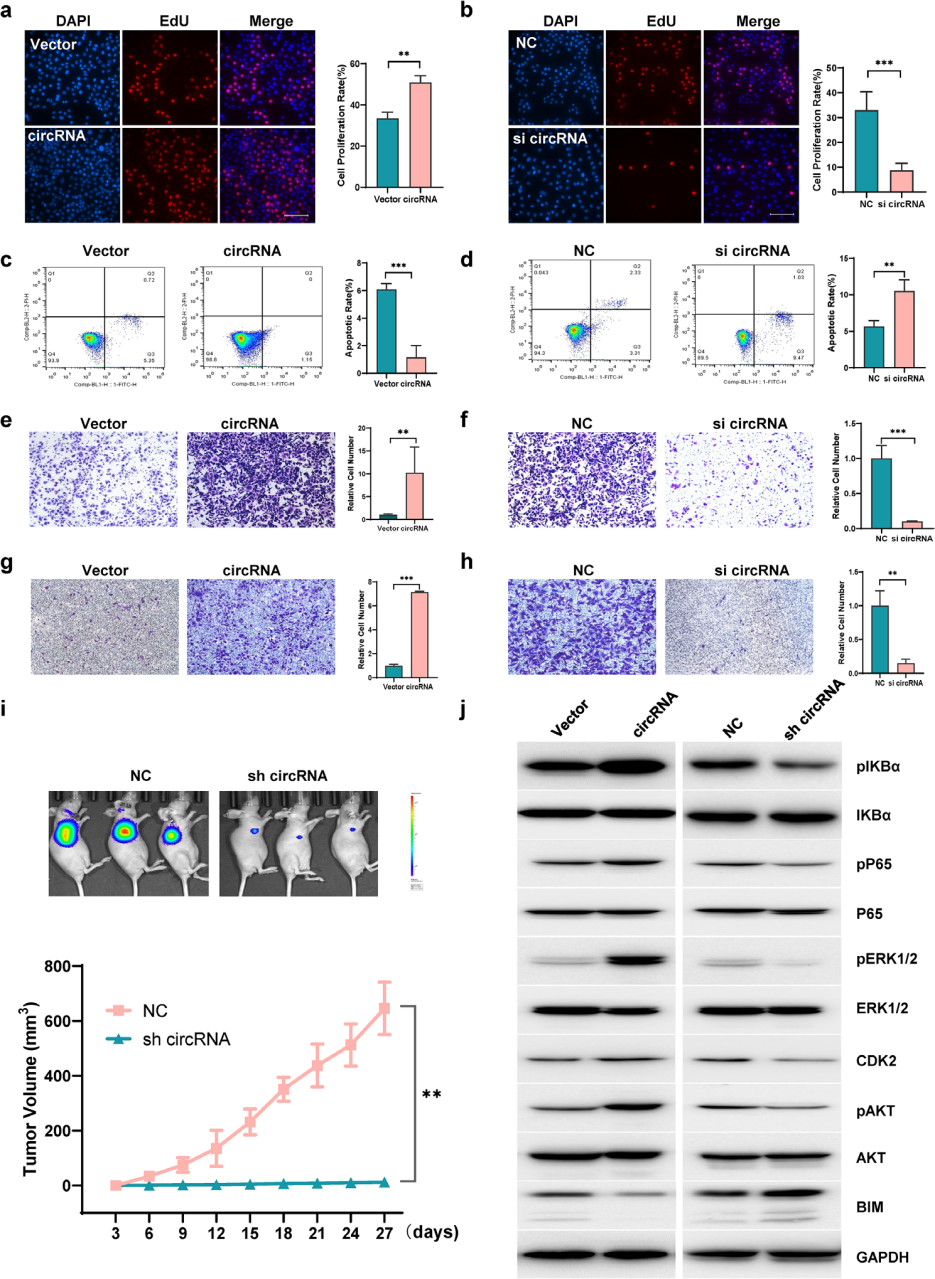
CircHERC1 promotes NCSLC cell proliferation, migration and invasion, and inhibits apoptosis. (**a**) EdU assay to detect the proliferation of NCI-H3255 cells stably overexpressing circHERC1. **p < 0.01. The scale bar is 25 μm. (**b**) EdU assay to detect the proliferation of NCI-H3255 cells with stable knockdown of circHERC1. ***p < 0.001. The scale bar is 25 μm. (**c**) Flow cytometry to detect apoptosis of NCI-H3255 cells stably overexpressing circHERC1. ***p < 0.001. (**d**) Flow cytometry to detect apoptosis of NCI-H3255 cells with stable knockdown of circHERC1. **p < 0.01. (**e, g**) Transwell assay to detect cell migration (e) and invasion (g) of NCI-H3255 cells stably overexpressing circHERC1. **p < 0.01, ***p < 0.001. (**f, h**) Transwell assay to detect cell migration (f) and invasion (h) of NCI-H3255 cells with stale knockdown of circHERC1. **p < 0.01, ***p < 0.001. (**i**) Representative images of tumor xenograft mouse models injected with NCI-H3255 cells with stable knockdown of circHERC1 and NCI-H3255 cells stably transfected with a scramble shRNA vector as negative control (top). The tumor volumes of mice in the sh-circHERC1 and control groups are shown(bottom). **p < 0.01. (**j**) Western blot analysis of the MAPK/ERK, NF-κB and PI3K/AKT pathways in NCI-H3255 cells with stable knockdown and overexpression of circHERC1

To study the role of circHERC1 in cancer progression, we first tested the endogenous expression of circHERC1 in lung cancer cell lines, human peripheral blood mononuclear cells (PBMC), human astrocytes (HA), human pericytes (PA) and human umbilical vein endothelial cells (HUVEC) (Fig. S1e). CircHERC1 was mainly expressed in lung cancer cells, so we focus on lung cancer cells. Two lung cancer cell lines (NCI-H3255 and A549) were chosen for subsequent experiments due to the highest circHERC1 expression levels among the five lung cancer cell lines. Furthermore, we constructed a circHERC1 overexpression plasmid, and confirmed that circHERC1 was overexpressed accurately and efficiently in NSCLC cells (Fig. S2a-b). For circHERC1 knockdown, siRNAs specifically targeting the backsplice junction region were designed (Fig. S2c-d). We investigated cell proliferation, apoptosis, migration and invasion after circHERC1 knockdown and overexpression in vitro. EdU experiments, CCK8 experiments and colony formation experiments proved that circHERC1 overexpression significantly increased the proliferation ability of BEAS-2B and NCI-H3255 cells (Fig. 2a, Fig. S2e-f, Fig. S2i and Fig. S2k), while the results observed in circHERC1 knockdown cells were contrary to those of the control group both in A549 and NCI-H3255 cells (Fig. 2b, Fig. S2g-h, Fig. S2j, and Fig. S2l). In the apoptosis detection experiments, circHERC1 overexpression inhibited apoptosis (Fig. 2c), while circHERC1 knockdown promoted apoptosis (Fig. 2d). Likewise, we found that upregulation of circHERC1 promoted the migration and invasion abilities of lung cancer cells through transwell and wound healing assays (Fig. 2e and g, Fig. S3a, Fig. S3c, Fig. S3e and Fig. S3g), whereas downregulation of circHERC1 exerted the opposite effect (Fig. 2f and h, Fig. S3b, Fig. S3d, Fig. S3f and Fig. S3h). Taken together, these results show that circHERC1 significantly affects the proliferation, migration, invasion and apoptosis of lung cancer cells.

To explore the biological functions of circHERC1 in lung cancer in vivo, we subcutaneously inoculated NCI-H3255 cells with circHERC1 shRNA plasmid, NCI-H3255 cells with scrambled shRNA plasmid and NCI-H3255 cells into nude mice. Tumor growth in the mice injected with sh-circHERC1 cancer cells was significantly lower than that in the mice injected with cells transfected with scrambled shRNA (Fig. 2i and Fig. S3i). These in vivo findings in ectopic xenograft mouse models are consistent with the in vitro observations, and hence support that suppression of circHERC1 could inhibit the in vivo tumorigenicity of lung cancer cells. Aberrantly expressed circRNAs play important roles in mediating cancer progression by regulating the activity of a variety of signaling pathways, such as the MAPK/ERK, NF-κB, and PI3K/AKT pathways [28–33]. After knockdown and overexpression of circHERC1, the expression of pIKBα, pP65, pERK1/2, CDK2, pAKT and BIM was tested, and the results showed that overexpression of circHERC1 promoted the expression of pIKBα, pP65, pERK1/2, pAKT and CDK2 and inhibited the expression of BIM (Fig. 2j). With circHERC1 downregulation, the proteins pIKBα, pP65, pERK1/2, CDK2 and pAKT were effectively downregulated, while the expression of BIM was upregulated.

### CircHERC1 functions as a sponge of miR-142-3p in NSCLC cells

**Fig. 3:**
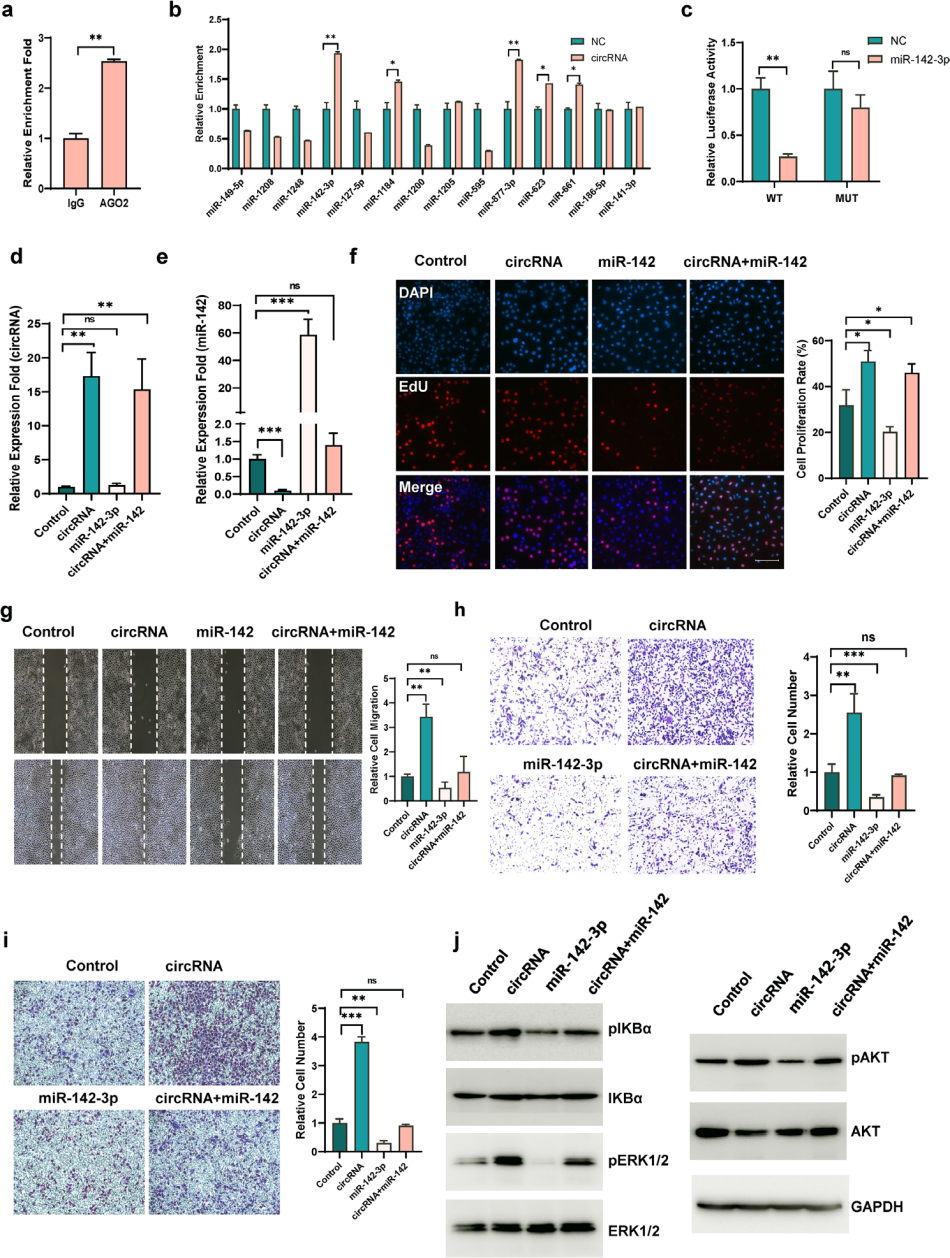
CircHERC1 directly binds miR-142 and regulates miR-142 expression in NSCLC cells. (**a**) qRT‒PCR for the expression of circHERC1 in NCI-H3255 cells after the pulldown of Ago2 by RIP assay. **p < 0.01. (**b**) qRT‒PCR of the candidate miRNA expression in circHERC1-overexpressing NCI-H3255 cell lysates pulled down and enriched with a biotinylated circHERC1-specific probe. *p < 0.05, **p < 0.01. (**c**) The relative luciferase activity of WT circHERC1 3^’^ UTR or mutant circHERC1 3’ UTR after transfection with miR-142 mimics in 293T cells. **p < 0.01. (**d, e**) qRT‒PCR was used to test the expression of circHERC1 and miR-142-3p in NCI-H3255 cells transfected with circHERC1 overexpression vector and miR-142 mimics. **p < 0.01, ***p < 0.001. (**f**) EdU analysis of the cell proliferation ability of NCI-H3255 cells transfected with the control, the circHERC1 overexpression vector, or miR-142 mimics and cotransfected with the circHERC1 overexpression vector and miR-142 mimics. *p < 0.05. The scale bar is 25 μm. (**g**) Wound healing assay to detect cell migration ability in NCI-H3255 cells transfected with the control, the circHERC1 overexpression vector, or miR-142 mimics and cotransfected with the circHERC1 overexpression vector and miR-142 mimics. **p < 0.01. (**h, i**) Transwell assay to detect cell migration (h) and invasion (i) of NCI-H3255 cells transfected with the control, the circHERC1 overexpression vector, or miR-142 mimics and cotransfected with the circHERC1 overexpression vector and miR-142 mimics. **p < 0.01, ***p < 0.001. (**j**) Western blot analysis of the MAPK/ERK, NF-κB and PI3K/AKT pathways in NCI-H3255 cells transfected with the control, the circHERC1 overexpression vector, or miR-142 mimics and cotransfected with the circHERC1 overexpression vector and miR-142 mimics

Considering that circHERC1 was mainly localized in the cytoplasm, we speculated that circHERC1 was involved in the progression of lung cancer through a miRNA sponge mechanism. Since Argonaute2 (AGO2) is an important mediator of circRNA-miRNA interactions [34, 35], an RNA immunoprecipitation (RIP) assay showed that circHERC1 was significantly enriched by the AGO2 protein in NCI-H3255 and A549 cells (Fig. 3a and Fig. S4a). This result suggested that circHERC1 may act as a miRNA sponge to participate in the AGO2-mediated competing endogenous RNA (ceRNA) mechanism of action. To investigate the downstream target miRNAs, we used three target prediction programs: CircInteractome (https://circinteractome.nia.nih.gov/), CSCD (http://gb.whu.edu.cn/CSCD/) and CircBank [36, 37] (Fig. S4b). Among the predicted miRNAs, we chose the 14 miRNAs most highly ranked as the candidate miRNAs. RNA pull-down experiments using biotinylated circHERC1 probes showed that miR-142-3p markedly enriched by the circHERC1 probe but not the scrambled oligonucleotide probe in A549 and NCI-H3255 cells (Fig. 3b and Fig. S4c). Then, we tested the expression of these 14 miRNAs in circHERC1 overexpressing and circHERC1 knockdown cells, and qRT‒PCR data showed that the miRNA miR-142-3p was downregulated after circHERC1 overexpression (Fig. S4d-e) and upregulated after circHERC1 knockdown (Fig. S4f-g). Sequences of circHERC1 incorporating the wild-type and mutant-type binding sites of miR-142-3p were synthesized and cloned into the dual-luciferase reporter vector (Fig. S4h). The activity signals of Renilla and firefly luciferase were detected by applying the Dual-Luciferase Report Gene Assay System. Mimics of mir-142-3p reduced luciferase activity in 293T cells transfected with the WT circHERC1 reporter, but not the MUT circHERC1 reporter (Fig. 3c). Active MAPK/ERK and NF-κB expression at the protein level was significantly reduced in lung cancer cells transfected with miR-142-3p mimics, while active MAPK/ERK and NF-κB expression was increased in NCI-H3255 cells transfected with miR-142-3p inhibitor (Fig. S4i-j, Fig. S4m). As expected, the expression of miR-142 in plasma was lower in patients with LUSC and LUAD than in cancer-free patients (Fig. S4k). In particular, the expression of miR-142 was extremely lower in plasma exosomes from late-stage lung cancer patients than those from early-stage lung cancer patients and cancer-free patients (Fig. S4l). To further investigate the functional interaction of circHERC1 and miR-142-3p in lung cancer cells, several rescue experiments were performed by cotransfection of miR-142-3p mimics and circHERC1 overexpression vector (Fig. 3d-e). The results showed that miR-142-3p overexpression eliminated the promoting effects of circHERC1 overexpression on cell migration and invasion (Fig. 3g-i), but had no effect on cell proliferation (Fig. 3f). Western blot experiments showed that circHERC1 affected the downstream MAPK/ERK and NF-κB signaling pathways through the miR-142-3p axis, but not the PI3K/ATK signaling pathway (Fig. 3j). These results suggest that circHERC1 acts as a functional sponge for miR-142-3p in lung cancer cells.

### HMGB1 is a functional target of miR-142-3p

**Fig. 4:**
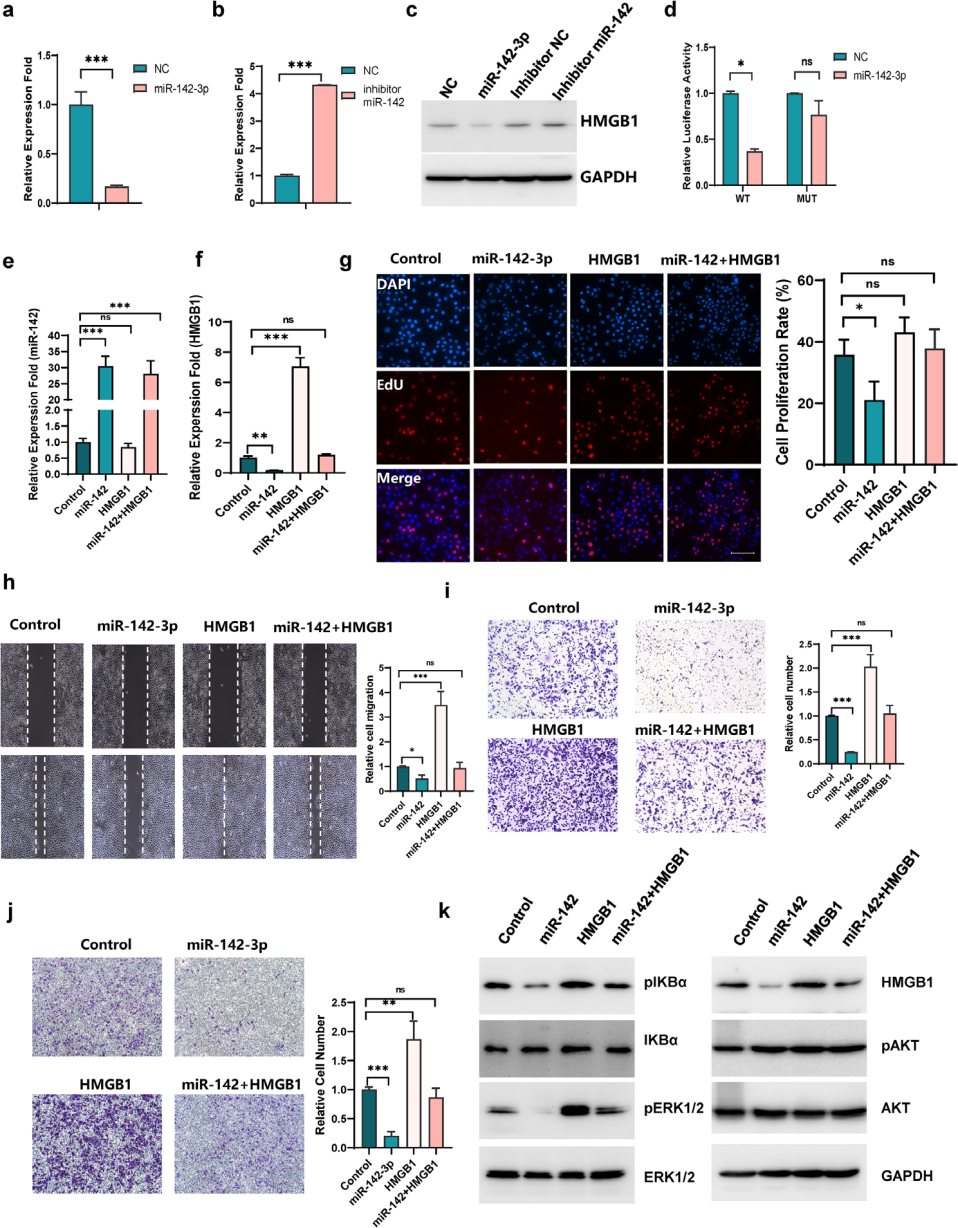
HMGB1 is a functional target of miR-142-3p. (**a, b**) qRT‒PCR analysis of HMGB1 expression in NCI-H3255 cells after transfection with miR-142-3p mimics or a miR-142-3p inhibitor. ***p < 0.001. (**c**) Western blot analysis of HMGB1 in NCI-H3255 cells after transfection with miR-142-3p mimics or a miR-142-3p inhibitor. (**d**) Relative luciferase activity of the WT HMGB1 3’ UTR or HMGB1 3’ UTR with mutation of potential binding sites after transfection with miR-142 mimics in 293T cells. *p < 0.05. (**e, f**) qRT‒PCR to test the expression levels of miR-142-3p and HMGB1 in NCI-H3255 cells transfected with the control, miR-142 mimics, or the HMGB1 overexpression vector and cotransfected with miR-142 mimics and the HMGB1 overexpression vector. **p < 0.01, ***p < 0.001. (**g**) EdU analysis of the cell proliferation ability of NCI-H3255 cells transfected with the control, miR-142 mimics, the HMGB1 overexpression vector and cotransfected with miR-142 mimics and the HMGB1 overexpression vector. *p < 0.05. The scale bar is 25 μm. (**h**) Wound healing assays to detect cell migration ability in NCI-H3255 cells transfected with the control, miR-142 mimics, or the HMGB1 overexpression vector and cotransfected with miR-142 mimics and the HMGB1 overexpression vector. *p < 0.05, ***p < 0.001. (**i, j**) Transwell assay to detect cell migration (i) and invasion (j) of NCI-H3255 cells transfected with the control, miR-142 mimics, or the HMGB1 overexpression vector respectively and cotransfected with miR-142 mimics and the HMGB1 overexpression vector. **p < 0.01, ***p < 0.001. (**k**) Western blot analysis of HMGB1 and the MAPK/ERK, NF-κB and PI3K/AKT pathways in NCI-H3255 cells transfected with the control, miR-142 mimics, or the HMGB1 overexpression vector and cotransfected with miR-142 mimics and the HMGB1 overexpression vector

To identify the potential target of miR-142-3p, three miRNA target gene databases (miRBD, miRBase and TargetScan) were exploited (Fig. S5a). We determined 15 genes for further study through research on lung cancer and its correlations, and qRT‒PCR was used to test the mRNA expression when circHERC1 was overexpressed and knocked down (Fig. S5b-c). Among the selected genes, HMGB1, a well-known tumor activator that promotes the metastasis of non-small cell lung cancer through NF-κB [38], plays a corresponding role in colorectal cancer by regulating ERK1/2 signaling [39], and is upregulated in human lung cancer. We found that HMGB1 was positively correlated with the expression of circHERC1 (Fig. 4a-b). qRT‒PCR and western blot analysis showed that HMGB1 expression at both the mRNA and protein levels was significantly reduced in lung cancer cells transfected with miR-142-3p mimics, while HMGB1 expression was increased in cells transfected with miR-142-3p inhibitor (Fig. 4a-c). HMGB1 displayed a significantly higher expression level in lung tumor tissues compared with the adjacent tissues (Fig. S5d). The TCGA database (https://tcga-data.nci.nih.gov/tcga/) was used to analyze differentially expressed miR-142-3p and HMGB1 in lung cancer patients. Patients with higher expression levels of HMGB1 tended to have shorter survival times, while patients with higher expression levels of miR-142-3p tended to have longer survival times (Fig. S5e, and Fig.S5h). To further elucidate the interaction between miR-142-3p and the HMGB1 3′UTR, a luciferase assay was subsequently performed in 293T cells. The pGL3- HMGB1-3′UTR vector or pGL3 basic vector was cotransfected with miR-142-3p mimics or the negative control into cells, and the luciferase activity was detected using a Dual-Luciferase Assay System. Cotransfection with miR-142-3p mimics significantly decreased the firefly luciferase activity of the HMGB1-3′UTR reporter but not that of the mutant HMGB1-3′UTR reporter (Fig. 4d and Fig. S5i). In summary, these findings indicate that HMGB1 is a direct target of miR-142-3p.

To further investigate the functional interaction of HMGB1 and miR-142-3p in lung cancer cells, several rescue experiments were performed by co-transfection with miR-142-3p mimics and a HMGB1 overexpression vector (Fig. 4e-f). The results showed that miR-142-3p, by targeting HMGB1, eliminated the promoting effects of circHERC1 overexpression on cell migration and invasion (Fig. 4h-j), but not on cell proliferation (Fig. 4g). Western blot experiment showed that HMGB1 and miR-142-3p affected the downstream MAPK/ERK and NF-κB signaling pathways, and this effect could be rescued by cotransfection with HMGB1 and miR-142-3p. but had no effect on the PI3K/ATK signaling pathway (Fig. 4k).

### CircHERC1 upregulates HMGB1 expression by sponging miR-142b-3p

**Fig. 5:**
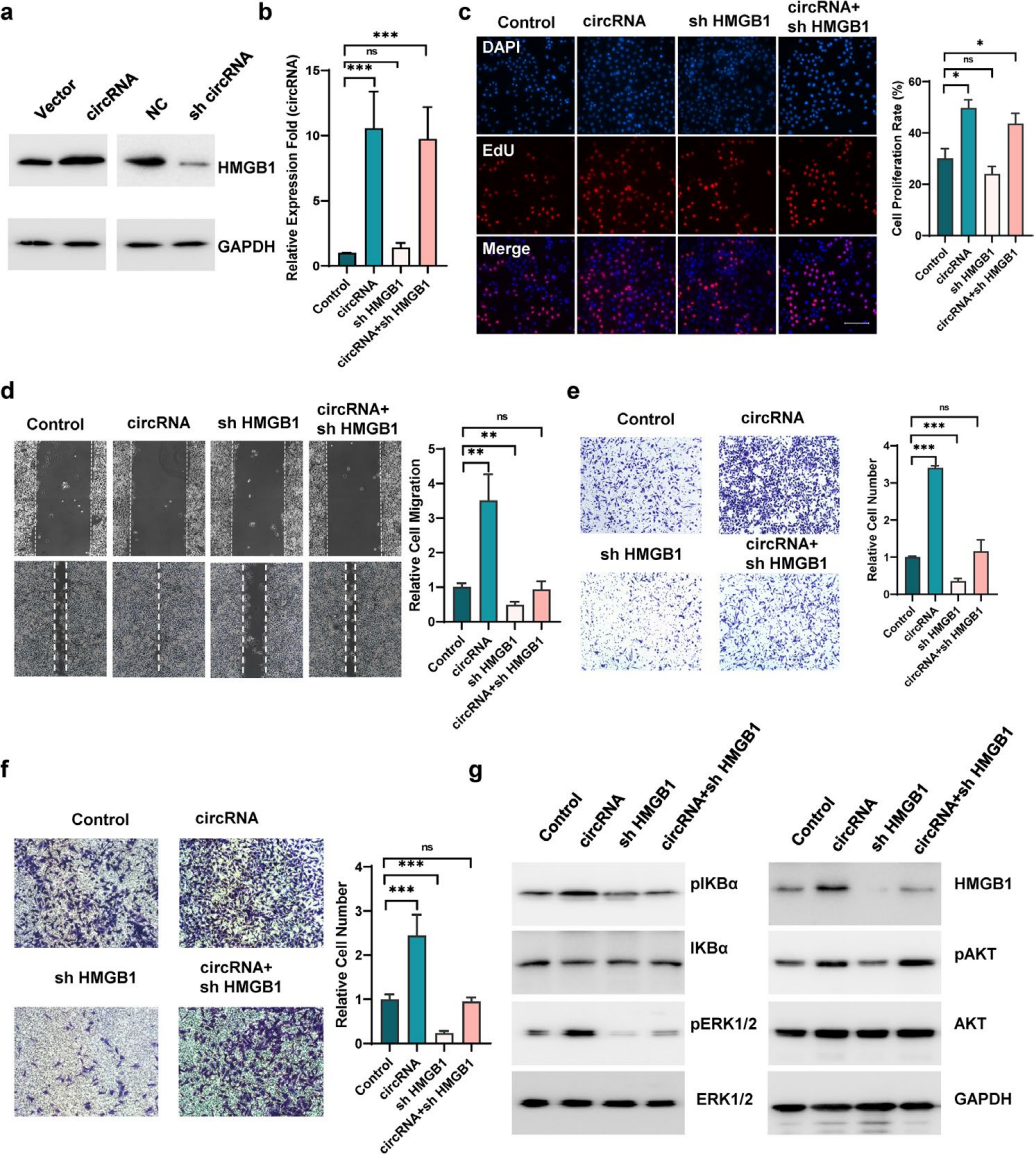
HMGB1 reversed circHERC1 upregulation-induced cell motility in lung cancer cells. (**a**) Western blot analysis of HMGB1 expression in NCI-H3255 cells with circHERC1 overexpression or knockdown. (**b**) qRT‒PCR to determine the level of circHERC1 in NCI-H3255 cells transfected with the control, a circHERC1 overexpression vector, or an HMGB1 knockdown vector and cotransfected with the circHERC1 overexpression vector and the HMGB1 knockdown vector. ***p < 0.001. (**c**) EdU analysis of the cell proliferation ability of NCI-H3255 cells transfected with the control, a circHERC1 overexpression vector, or an HMGB1 knockdown vector and cotransfected with the circHERC1 overexpression vector and the HMGB1 knockdown vector. *p < 0.05. The scale bar is 25 μm. (**d**) Wound healing assays to detect cell migration ability of NCI-H3255 cells transfected with the control, a circHERC1 overexpression vector, or an HMGB1 knockdown vector and cotransfected with the circHERC1 overexpression vector and the HMGB1 knockdown vector. **p < 0.01, ***p < 0.001. (**e, f**) Transwell assay to detect cell migration (e) and invasion (f) in NCI-H3255 cells transfected with the control, a circHERC1 overexpression vector, or an HMGB1 knockdown vector and cotransfected with the circHERC1 overexpression vector and the HMGB1 knockdown vector. ***p < 0.001. (**g**) Western blot analysis of HMGB1 and the MAPK/ERK, NF-κB and PI3K/AKT pathways in NCI-H3255 cells transfected with the control, a circHERC1 overexpression vector, or an HMGB1 knockdown vector and cotransfected with the circHERC1 overexpression vector and the HMGB1 knockdown vector

qRT‒PCR and western blot analysis showed HMGB1 expression was upregulated when circHERC1 was overexpressed in NCI-H3255 cells (Fig. 5a and Fig. S6a-b), while it was downregulated when circHERC1 was knocked down (Fig. 5b and Fig. S6c-d). To further investigate the functional interaction of circHERC1 and HMGB1 in lung cancer cells, EdU experiments, CCK8 experiments and apoptosis detection experiments were performed, which showed that sh-HMGB1 did not eliminate the promoting effects of circHERC1 overexpression on cell proliferation (Fig. 5c and Fig. S6e), and the inhibition of apoptosis (Fig. S6f). Transwell and wound healing assays showed that sh-HMGB1 eliminated the promoting effects of circHERC1 overexpression on cell migration and invasion (Fig. 5d-f). In lung cancer tissues, HMGB1 was positively correlated with circHERC1 (Fig. S6g). Western blot experiments showed that circHERC1 affected the downstream MAPK/ERK and NF-κB signaling pathways through the miR-142-HMGB1 axis, but had no effect on the PI3K/ATK signaling pathway (Fig. 5g). These results suggest that circHERC1 functions as a ceRNA for miR-142-3p and regulates the expression and activity of HMGB1. The circHERC1-miR-142-3p-HMGB1 axis regulates cell migration and invasion in lung cancer cells, but the axis has little effect on cell viability regulation. Therefore, there may be other mechanisms involved in regulating the function of circHERC1.

### CircHERC1 binds FOXO1 and sequesters it in the cytoplasm to promote cell proliferation by feedback AKT activation

**Fig. 6:**
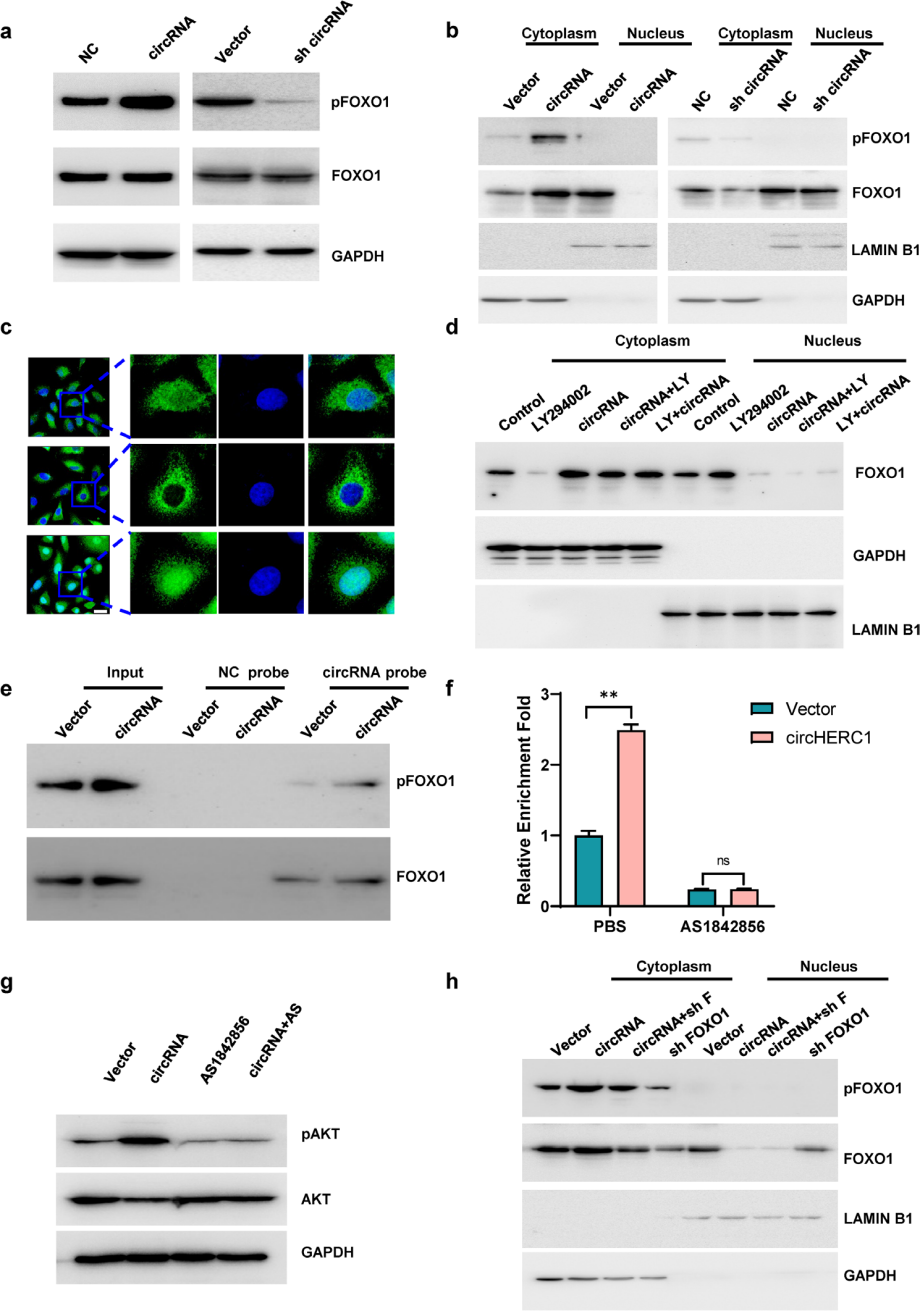
CircHERC1 binds FOXO1 and sequesters it in the cytoplasm. (**a**) Western blot analysis of pFOXO1 and FOXO1 in NCI-H3255 cells with circHERC1 overexpression or knockdown. (**b**) Western blot analysis of pFOXO1 and FOXO1 in the cytoplasm and nucleus of NCI-H3255 cells with circHERC1 overexpression or knockdown. (**c**) IF assays to detect FOXO1 subcellular localization in A549 cells after alteration of the expression of circHERC1 The scale bars are 15 μm. (**d**) Western blot analysis of FOXO1 in the cytoplasm and nucleus in NCI-H3255 cells transfected with the control vector, control vector treated with LY2940002, circHERC1 overexpression vector, and circHERC1 overexpression vector treated with LY2940002, or cells pretreated with LY2940002 transfected with circHERC1 overexpression vector. (**e**) RNA pulldown assays to evaluate the interaction between circHERC1 and pFOXO1 or FOXO1 using biotinylated probes against circHERC1. (**f**) RIP assays to evaluate the interaction between circHERC1 and FOXO1 after alteration of the expression of circHERC1 and treatment with AS1842856 using a FOXO1 antibody. **p < 0.01. (**g**) Western blot analysis of pAKT in NCI-H3255 cells with circHERC1 overexpression treatment with AS1842856. (**h**) Western blot analysis of pFOXO1 and FOXO1 in the cytoplasm and nucleus in NCI-H3255 cells transfected with the control vector, circHERC1 overexpression vector, or FOXO1 knockdown vector and cotransfected with the circHERC1 overexpression vector and FOXO1 knockdown vector

CircHERC1-miR-142-HMGB1 axis had no effect on cell proliferation and apoptosis, while LY294002, a PI3K inhibitor, attenuated the cell proliferation promotion and the apoptosis inhibition induced by circHERC1 overexpression (Fig. S7). We then focus on the PI3K/ATK signaling pathway, in which the transcriptional activity of forkhead box transcription factor class O (FOXO) proteins, the key downstream targets of AKT signaling, can result in a variety of cellular outcomes depending on the cell type and activating stimulus [40]. We first analyzed the expression of FOXO1 in 16 lung cancer tissues with adjacent tissues by qRT-PCR, and found that the expression of FOXO1 in lung cancer tissues was lower than that in adjacent tissues (Fig. S8a). From TCGA data, we also found that FOXO1 was down-regulated in lung cancer tissues (Fig. S8b-g). ROC curve analysis indicated a relatively high diagnostic value of FOXO1 for LUAD, LUSC and lung cancers (Fig. S8h-j). These results implied that FOXO1 might be the major target to regulate lung cancer cell survival. Overexpression of circHERC1 led to increased phosphorylation of FOXO1, while inhibition of circHERC1 decreased the phosphorylation of FOXO1 (Fig. 6a). We found that circHERC1 overexpression caused FOXO1 to almost translocated to the cytosol both by western blot analysis of nuclear and cytoplasm fractions (Fig. 6b) and IF assays (Fig. 6c and Fig. S9a). By combining the FAM-labeled circHERC1 FISH assay and IF assay, we found that FOXO1 nuclear exclusion mainly occurred in circHERC1 overexpressing cells (Fig. S9b). Activation of PI3K/AKT signaling leads to acute translocation of FOXO1 out of the nucleus [40]. To verify whether FOXO1 translocation is caused by AKT activation or circHERC1 overexpression, circHERC1 was overexpressed in LY294002 pretreated NCI-H3255 cells, and the results showed that FOXO1 cytosol accumulation was independent of AKT phosphorylation (Fig. 6d, and Fig. S10a-b). As circHERC1 predominantly localized in the cytoplasm (Fig. 1g), we hypothesized that circHERC1 could bind FOXO1 and facilitate the sequestration of FOXO1 in the cytosol. We performed RIP and RNA pulldown assays to verify the interaction with FOXO1 and circHERC1, and found that FOXO1 and circHERC1 could combine with each other (Fig. 6e-f and Fig. S11a-b). FOXO1 inhibitor, AS1842586, could disrupt the interaction between FOXO1 and circHERC1 (Fig. 6f and Fig. S11b), and the dissociation could restore the phosphorylation status of AKT induced by circHERC1 overexpression (Fig. 6g). AS1842586 also attenuated the cell proliferation promotion and the apoptosis inhibition induced by circHERC1 overexpression (Fig. S12). Previous evidence has shown that FOXO1 can feedback activate AKT, and the feedback activation of AKT maintains growth factor-responsive AKT [41, 42]. Knockdown of FOXO1 in lung cancer cells downregulated the activation of AKT, and the AKT activation was not restored by circHERC1 overexpression (Fig. S13a). Cytosolic FOXO1, not whole-cell FOXO1, seemed to activate AKT in a feedback manner. By western blot analysis of the nuclear and cytoplasmic fractions, we found that cytosolic FOXO1 was positively correlated with the activation of AKT (Fig. 6h). FOXO1 cytosol accumulation, sequestered by circHERC1, feedback activated AKT to promote cell proliferation (Fig. S13b), while FOXO1 nuclear exclusion inhibited apoptosis (Fig. S13c). On the other hand, circHERC1 overexpressing lung cancer cells lost the reactivity to EGF (Fig. S14). These results further confirm that circHERC1 binds FOXO1 and sequesters it in the cytoplasm to promote cell proliferation by feedback-activating AKT.

### Inhibition of HMGB1 and FOXO1 expression can restore the influence of circHERC1 on cell proliferation, apoptosis, migration and invasion

**Fig. 7:**
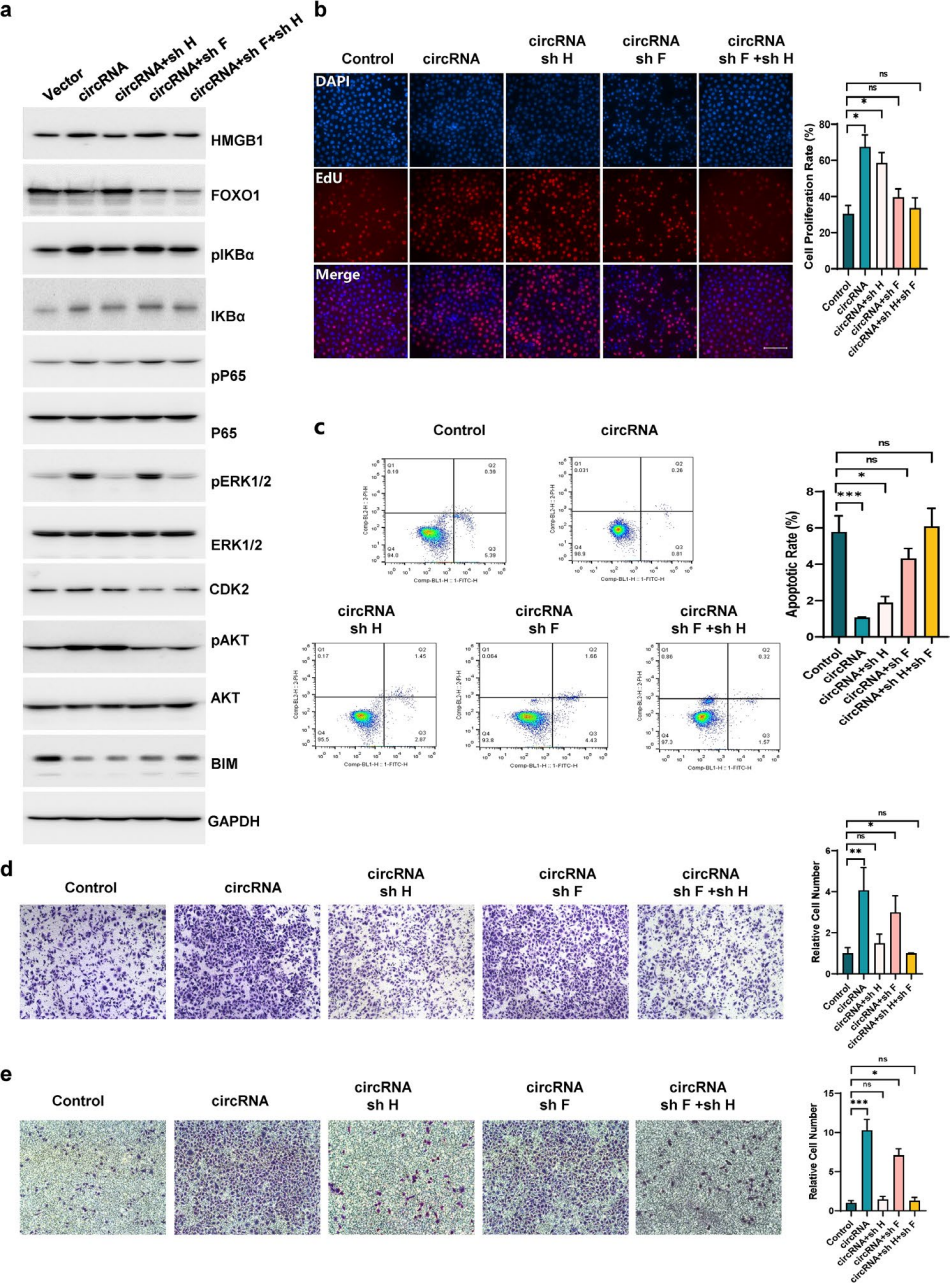
Inhibition of HMGB1 and FOXO1 expression attenuates the influence of circHERC1 on cell proliferation, apoptosis, migration and invasion. (**a**) Western blot analysis of HMGB1, FOXO1 and the MAPK/ERK, NF-κB and PI3K/AKT pathways in NCI-H3255 cells transfected with the control vector, or the circHERC1 overexpression vector and cotransfected with the circHERC1 overexpression vector and the HMGB1 knockdown vector; the circHERC1 overexpression vector and the FOXO1 knockdown vector; or the circHERC1 overexpression vector, the HMGB1 knockdown vector, and the FOXO1 knockdown vector. (**b**) EdU analysis of the cell proliferation ability in NCI-H3255 cells transfected with the control vector, or the circHERC1 overexpression vector and cotransfected with the circHERC1 overexpression vector and the HMGB1 knockdown vector; the circHERC1 overexpression vector and the FOXO1 knockdown vector; or the circHERC1 overexpression vector, the HMGB1 knockdown vector, and the FOXO1 knockdown vector. *p < 0.05. The scale bar is 25 μm. (**c**) Flow cytometry to detect apoptosis of NCI-H3255 cells transfected with the control vector, or the circHERC1 overexpression vector and cotransfected with the circHERC1 overexpression vector and the HMGB1 knockdown vector; the circHERC1 overexpression vector and the FOXO1 knockdown vector; or the circHERC1 overexpression vector, the HMGB1 knockdown vector, and the FOXO1 knockdown vector. *p < 0.05, ***p < 0.001. (**d, e**) Transwell assay to detect cell migration (d) and invasion (e) of NCI-H3255 cells transfected with the control vector, or the circHERC1 overexpression vector and cotransfected with the circHERC1 overexpression vector and the HMGB1 knockdown vector; the circHERC1 overexpression vector and the FOXO1 knockdown vector; or the circHERC1 overexpression vector, the HMGB1 knockdown vector, and the FOXO1 knockdown vector. *p < 0.05, **p < 0.01, ***p < 0.001

Dysregulation of circHERC1 contributed to tumorigenesis of NSCLC. To verify the function of circHERC1 through FOXO1 and HMGB1, many rescue experiments were performed. FOXO1 knockdown attenuated the activation of AKT and CDK2 and the inhibition of BIM caused by FOXO1 cytosolic accumulation and nuclear exclusion due to circHERC1 overexpression (Fig. 7a). HMGB1 knockdown reversed the activation of the MAPK/ERK and NF-κB signaling pathways induced by circHERC1 overexpression (Fig. 7a). FOXO1 and HMGB1 knockdown simultaneously restored all of the above signaling pathways to normal levels (Fig. 7a). We also conducted EdU, apoptosis and transwell assays to detect the rescue of FOXO1 and HMGB1 knockdown on cell proliferation, apoptosis, migration and invasion in circHERC1 overexpressing lung cancer cells. The results showed that FOXO1 knockdown rescued cell viability (Fig. 7b-c) and HMGB1 knockdown rescued cell motility (Fig. 7d-e) from the effects of circHERC1.

### CircHERC1 can promote tumor proliferation and metastasis in vivo

**Fig. 8:**
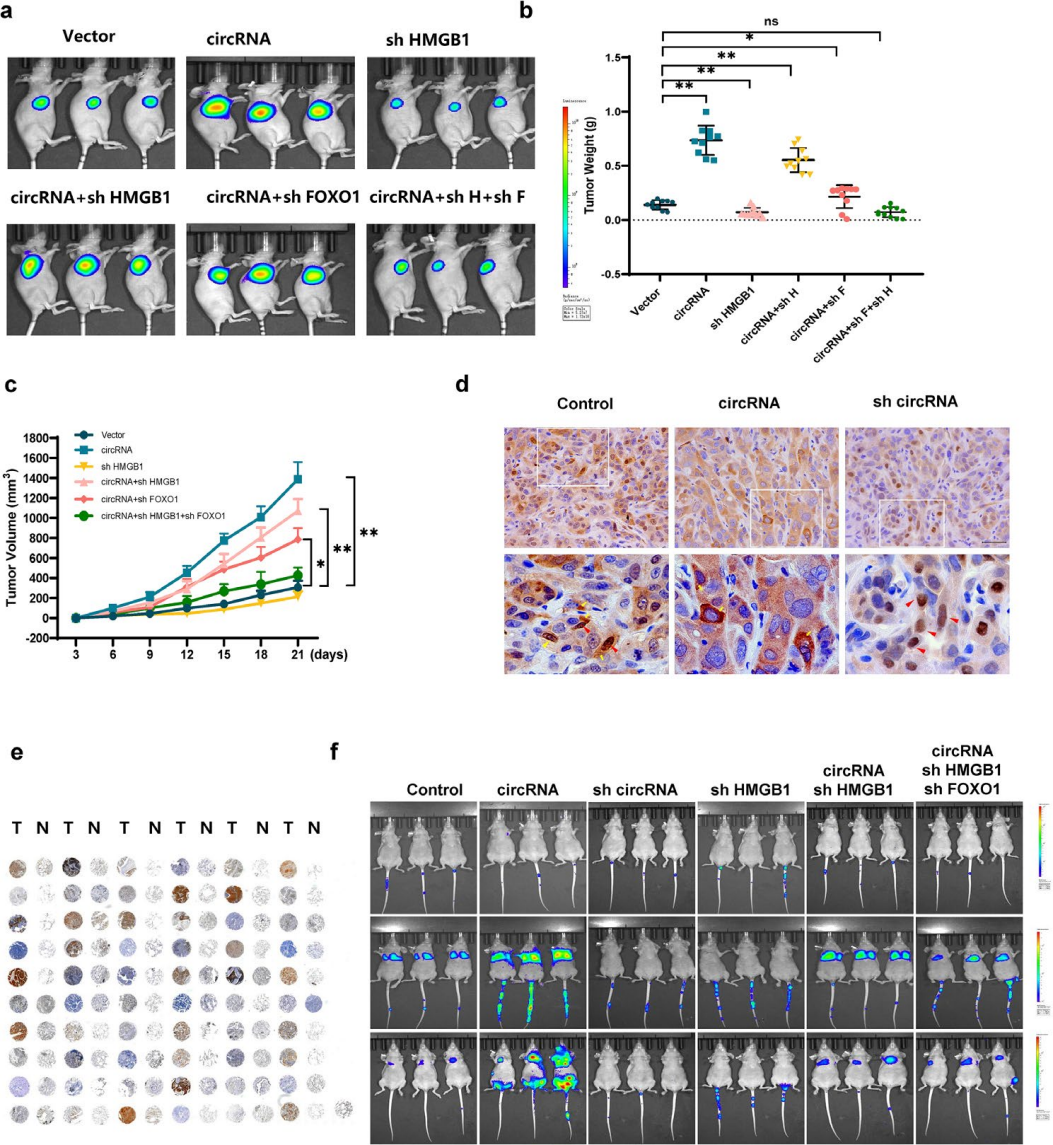
CircHERC1 promotes tumor proliferation and metastasis in vivo. (**a**) Representative images of tumor xenograft mouse models injected with NCI-H3255 cells transfected with the control vector, the circHERC1 overexpression vector, or the HMGB1 knockdown vector and cotransfected with the circHERC1 overexpression vector and the HMGB1 knockdown vector; the circHERC1 overexpression vector and the FOXO1 knockdown vector; or the circHERC1 overexpression vector, the HMGB1 knockdown vector, and the FOXO1 knockdown vector. (**b**) Weights of xenograft tumors in mice injected with NCI-H3255 at the endpoint. *p < 0.05, **p < 0.01. (**c**) Growth curve of tumor volume. *p < 0.05, **p < 0.01. (**d**) IHC staining of FOXO1 in xenograft tumors. The scale bars are 25 μm. (**e**) IHC staining of HMGB1 on a lung cancer tissue microarray (R121Lu01, Bioaitech, China). (**f**) Representative images of nude mice injected with the treated-NCI-H3255 cells mentioned above through the tail vein

To explore the biological function of circHERC1 in vivo, we subcutaneously inoculated NCI-H3255 cancer cells with altered expression of circHERC1, HMGB1 or FOXO1, into the flanks of nude mice. In comparison with the control, in vivo imaging of mice showed that circHERC1 overexpression significantly increased the mean tumor volume, tumor weight and fluorescence intensity. HMGB1 knockdown inhibited tumor cell proliferation, but HMGB1 knockdown had little ameliorative effect on the tumor promotion caused by circHERC1 overexpression. The effect of circHERC1 overexpression was rescued when cells stably overexpressing circHERC1 were transfected with a HMGB1 knockdown plasmid and a FOXO1 knockdown plasmid (Fig. 8a-c). At the same time, the expression of FOXO1, HMGB1, pERK1/2, pIKBα and pATK in the tumor tissues of mice was verified by western blot analysis and immunohistochemistry, and the results were consistent with the above in vitro experimental results (Fig. 8d, Fig. S15, and Fig. S16). At the same time, we performed a tissues microarray by using patients’ samples and found that the expression level of HMGB1 in lung cancer tissues was considerably higher than that in para-cancerous tissues (Fig. 8e). To determine the effect of circHERC1 on tumor cell migration in vivo, a mouse model of tumor metastasis was established. In vivo imaging of mice showed that circHERC1 overexpression significantly promoted tumor metastasis. HMGB1 knockdown inhibited tumor metastasis, and HMGB1 knockdown attenuated tumor metastasis caused by circHERC1 overexpression (Fig. 8f). These in vivo findings in ectopic xenograft mouse models were consistent with the in vitro observations, and hence support that overexpression of circHERC1 can promote in vivo tumorigenicity of lung cancer cells via the miR-142-3p-HMGB1 axis and interaction with FOXO1.

## Discussion

Recently, circRNAs have become a hot topic in RNA research since they play key roles in numerous physiological and pathological processes of a wide variety of diseases [43]. In particular, circRNAs can regulate different molecular signaling pathways by acting as miRNA sponges, anchors for circRNA binding proteins (cRBPs), transcriptional regulators, and sources for translation of small proteins/peptides [44]. In this study, we found that expression levels of many circRNAs were significantly upregulated in lung cancer patient plasma through high-throughput sequencing. Among these circRNAs, circHERC1 was characterized as a lung cancer-associated circRNA derived from exons 22–27 of HERC1 that was formed a circRNA through back-splicing. Interestingly, circHERC1 was altered not only in plasma of lung cancer patients, but also in tissue and plasma exosome of lung cancer patients.

Elevated circHERC1 expression is frequently observed in lung tumor tissues and plasma exosomes of early-stage and late-stage lung cancer patients. CircHERC1 may be a cell-free circulating biomarker for lung cancer. Furthermore, we confirmed that circHERC1 has an important function in the pathogenesis of NSCLC since its dysregulation contributed to NSCLC cell growth, proliferation, migration, invasion and apoptosis. Moreover, the in vivo tumorigenicity of circHERC1 was confirmed by observation of ectopic xenograft mouse models. Therefore, the possible pathways by which circHERC1 might participate in the development and progression of NSCLC need to be investigated. We found that overexpression of circHERC1 promoted the activation of AKT, ERK1/2 and IKBα which controlled several intracellular signaling molecules for cell growth and survival, Moreover, overexpression of circHERC1 inhibited the expression of BIM that was intrinsically responsible for the initiation of apoptosis, whereas knockdown of circHERC1 had the opposite effects. The molecular mechanisms of circHERC1 in tumorigenesis need to be further investigated.

Previous studies have shown that the cytoplasmic localization of circRNA is closely related to the sponging effect of miRNA [45, 46]. In this study, we found that circHERC1 predominantly localized in the cytoplasm. RNA pulldown with a probe against the back-spliced junction of circHERC1 displayed enrichment of miR-142-3p. Previous research has shown that miR-142-3p suppresses cell proliferation, migration and invasion through inhibition of NR2F6 in lung adenocarcinoma and functions as a potential tumor suppressor in NSCLC [47, 48]. Rescue experiments showed that miR-142-3p can attenuate the effects of circHERC1 overexpression on migration and invasion, but not proliferation. Mechanistically, circHERC1 functions as a sponge to reduce the expression of miR-142-3p, a negative regulator of lung cancer progression and metastasis. The activation of ERK1/2 and IKBα, not AKT, altered by circHERC1 overexpression could be reversed by miR-142-3p. Next, we investigated the regulatory role of miR-142-3p in NSCLC cells and its impact on NSCLC tumorigenesis in vitro. Previous studies and our study have verified that HMGB1 is a direct target gene of miR-142-3p [49]. As a non-histone chromatin-associated protein, HMGB1 performs a pivotal function in various human diseases, including autoimmune diseases, neurodegenerative diseases and cancer. Overexpression of HMGB1 has been demonstrated in numerous types of cancer, including breast cancer, colorectal cancer, hepatocellular carcinoma and lung cancer. Rescue experiments showed that miR-142-3p can restore the effect of HMGB1 overexpression on migration and invasion. The expression of pERK1/2 and pIKBα induced by HMGB1 overexpression can be reversed by miR-142-3p.

Many studies have shown that circRNAs act as ceRNAs that disrupt the ceRNA-miRNA-mRNA axis [43]. We studied the functional interaction between HMGB1 and circHERC1 and found that circHERC1 functions as a ceRNA for miR-142-3p and regulates the expression and activity of HMGB1 without affecting cell proliferation and apoptosis. Therefore, there may be other mechanisms regulating the function of circHERC1. Because dysregulation of circHERC1 can induce the activation of AKT, the proliferation of NSCLC cells and the expression of pAKT cannot be affected by HMGB1 and miR-142-3p.

AKT mediates multiple critical signal transduction processes, including cell proliferation and survival, by phosphorylating downstream factors. FOXO1 has been found to be one of the most important substrates of AKT. In previous studies, AKT-mediated phosphorylation of FOXO1 led to the nuclear export and subsequent degradation of FOXO1 via the proteasome, which consequently inactivated the transcriptional activity of the protein [40, 50]. It has also been demonstrated that FOXO1 has transcriptional activity in the nucleus, where it can regulate the transcription of BIM and affect apoptosis [51, 52]. In this study, we performed RIP and RNA pulldown assays to study the binding between circHERC1 and the FOXO1 protein. RIP assays showed that circHERC1 was more enriched in anti-FOXO1 than in anti-IgG immunoprecipitates. RNA pulldown assays showed that biotinylated circHERC1 specific probes could pull down FOXO1 proteins, while the scramble probe could not. Further evidence showed circHERC1 bound FOXO1 and sequestered it in the cytoplasm to promote cell proliferation by feedback-activating AKT. CircHERC1 functioned as a proliferation activator and apoptosis inhibitor through FOXO1 translocation from the nucleus to the cytoplasm. Moreover, we analyzed the gene expression profile and corresponding clinical information of patients from The Cancer Genome Atlas (TCGA) database and found that HMGB1 was correlated with poor prognosis of NSCLC patients. The ROC curves of miR142-3p, HMGB1 and FOXO1 were plotted to validate the predictive effect of the prognostic signature indicating that circHERC1 may also serve as a prognostic biomarker for NSCLC.

The in vivo findings in ectopic xenograft mouse models further confirmed that the circHERC1 overexpression resulted in larger tumor volumes and stronger fluorescence than the control, while this promoting effect was attenuated by FOXO1 knockdown. In addition, knockdown of HMGB1 and FOXO1 simultaneously restored the changes in the tumor size, volume and fluorescence intensity in mice induced by circHERC1 overexpression.

There are still some limitations in this present study. As a diagnosis marker, a prospective and multisite lung cancer screening trial is needed to validate the diagnostic value of circHERC1.The relationship between circHERC1 and other molecules by using in vitro transcription and cyclization of circHERC1 need to be elucidated. It would be more accurate and meaningful to investigate the role of circHERC1 in clinical application for lung cancer treatment.

## Conclusion

Overall, for the first time, our data demonstrated that circHERC1 plays an oncogenic role in lung cancer development via miR-142-3p/HMGB1 axis which enhanced the expression of HMGB1 to activate the MAPK/ERK and NF-κB pathways by sponging miR-142-3p in cancer cells. The other underlying mechanism is that circHERC1 promotes cell proliferation and inhibits apoptosis by sequestering FOXO1 in the cytoplasm to regulate AKT activity and BIM transcription (Fig. S17). These help us better understand the mechanism of circHERC1 in lung cancer progression and provide a promising biomarker for lung cancer treatment.

### Electronic supplementary material

Below is the link to the electronic supplementary material.

Additional file 1: Supplementary Table 1. The RNA-seq results of all differentially expressed circRNAs in 9 plasma

Additional file 2: Figure S1. Oncogenic circRNA discovery and characterization of circHERC1 in NSCLC (a-e)

Additional file 3: Figure S2. CircHERC1 promotes NSCLC cell proliferation (a-l)

Additional file 4: Figure S3. CircHERC1 promotes NSCLC cell migration and invasion (a-i)

Additional file 5: Figure S4. CircHERC1directly binds miR-142 and regulates miR-142 expression in NSCLC cells(a-m)

Additional file 6: Figure S5. HMGB1 is a functional target of miR-142-3p(a-i)

Additional file 7: Figure S6. CircHERC1 upregulates HMGB1 expression by sponging miR-142 (a-g)

Additional file 8: Figure S7. CircHERC1 elevates cell viability through the PI3K/AKT pathway (a-b)

Additional file 9: Figure S8. Down-regulated expression of FOXO1 in lung cancers (a-m)

Additional file 10: Figure S9. FOXO1 accumulation in the cytoplasm in circHERC1 overexpressing cells (a-b)

Additional file 11: Figure S10. FOXO1 accumulation in the cytoplasm independent of AKT activation (a-b)

Additional file 12: Figure S11. CircHERC1 interacts with FOXO1 (a-b)

Additional file 13: Figure S12. CircHERC1 elevates cell viability by interacting with FOXO1 (a-b)

Additional file 14: Figure S13. Inhibition of FOXO1 expression attenuates the influence of circHERC1 on cell proliferation and apoptosis (a-c)

Additional file 15: Figure S14. Feedback AKT activation by FOXO1 leads to loss of reactivity to EGF (a-b)

Additional file 16: Figure S15. Western blot analysis of HMGB1, FOXO1, MAPK/ERK, IKBα and PI3K/AKT in xenograft tumors

Additional file 17: Figure S16. IHC staining of FOXO1, HMGB1, Ki67, pIKBα and pERK1/2 in xenograft tumors

Additional file 18: Figure S17. Schematic diagram illustrating the mechanism

Supplementary Material 19

## Data Availability

All materials underlying this study are available from the corresponding author on the basis of a material transfer agreement.

## Abbreviations

circRNA: Circular RNA
NSCLC: Non-small cell lung cancer
LUAD: Lung adenocarcinoma
LUSC: Lung squamous cell carcinoma
qRT-PCR: Quantitative reverse transcription polymerase chain reaction
RIP: RNA immunoprecipitation
FISH: Fluorescent in situ hybridization
ncRNAs: Noncoding RNA
miRNA: microRNA
AGO2: Argonaute-2
3’UTR: 3′-untranslated regions
EdU: 5-ethynyl-2’-deoxyuridine
CCK8: Cell count kit-8
IHC: Immunohistochemistry
HE: Hematoxylin and eosin
shRNA: Short hairpin RNA
siRNA: Small interfering RNA
HMGB1: High-mobility group box 1
MAPK: Mitogen-activated protein kinase
ERK: Extracellular-signal-regulated kinase
NF-κB: Nuclear factor-kappa B
IKBα: NF-kappa-B inhibitor alpha
PI3K: Phosphatidylinositol 3-kinase
AKT: Protein kinase B
FOXO1: Forkhead box O1
BIM: Bcl-2-like protein 11
