# Supplementary figures and images for: CircHERC1 promotes non-small cell lung cancer cell progression by sequestering FOXO1 in the cytoplasm and regulating the miR-142-3p-HMGB1 axis
Source: Mol Cancer. 2023 Nov 6;22:179. doi: 10.1186/s12943-023-01888-7 (PMC10626661; doi:10.1186/s12943-023-01888-7)

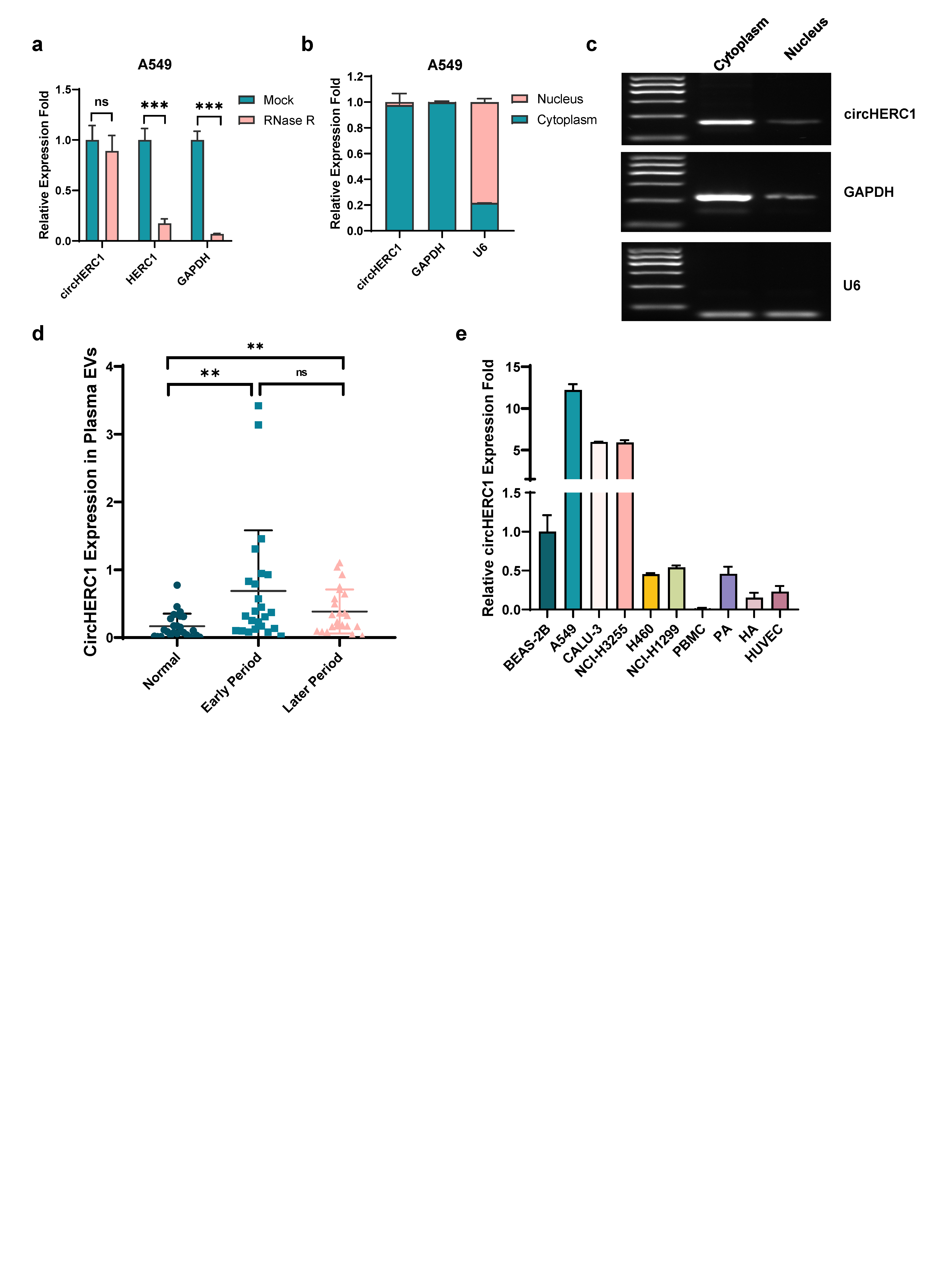

Supplement: Supplementary file 2 — Additional file 2: Figure S1. Oncogenic circRNA discovery and characterization of circHERC1 in NSCLC (a-e) [file 12943_2023_1888_MOESM2_ESM.png]

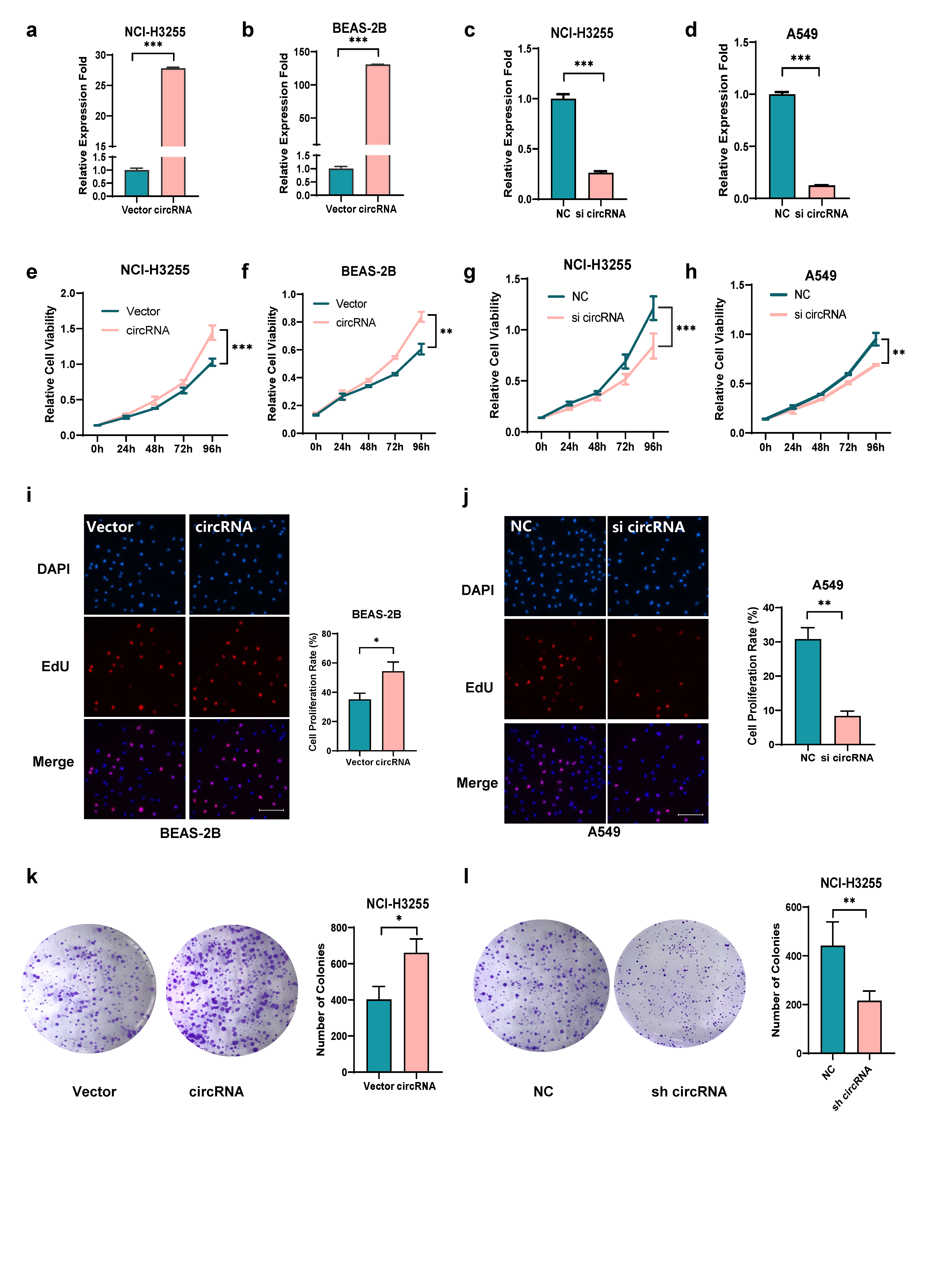

Supplement: Supplementary file 3 — Additional file 3: Figure S2. CircHERC1 promotes NSCLC cell proliferation (a-l) [file 12943_2023_1888_MOESM3_ESM.png]

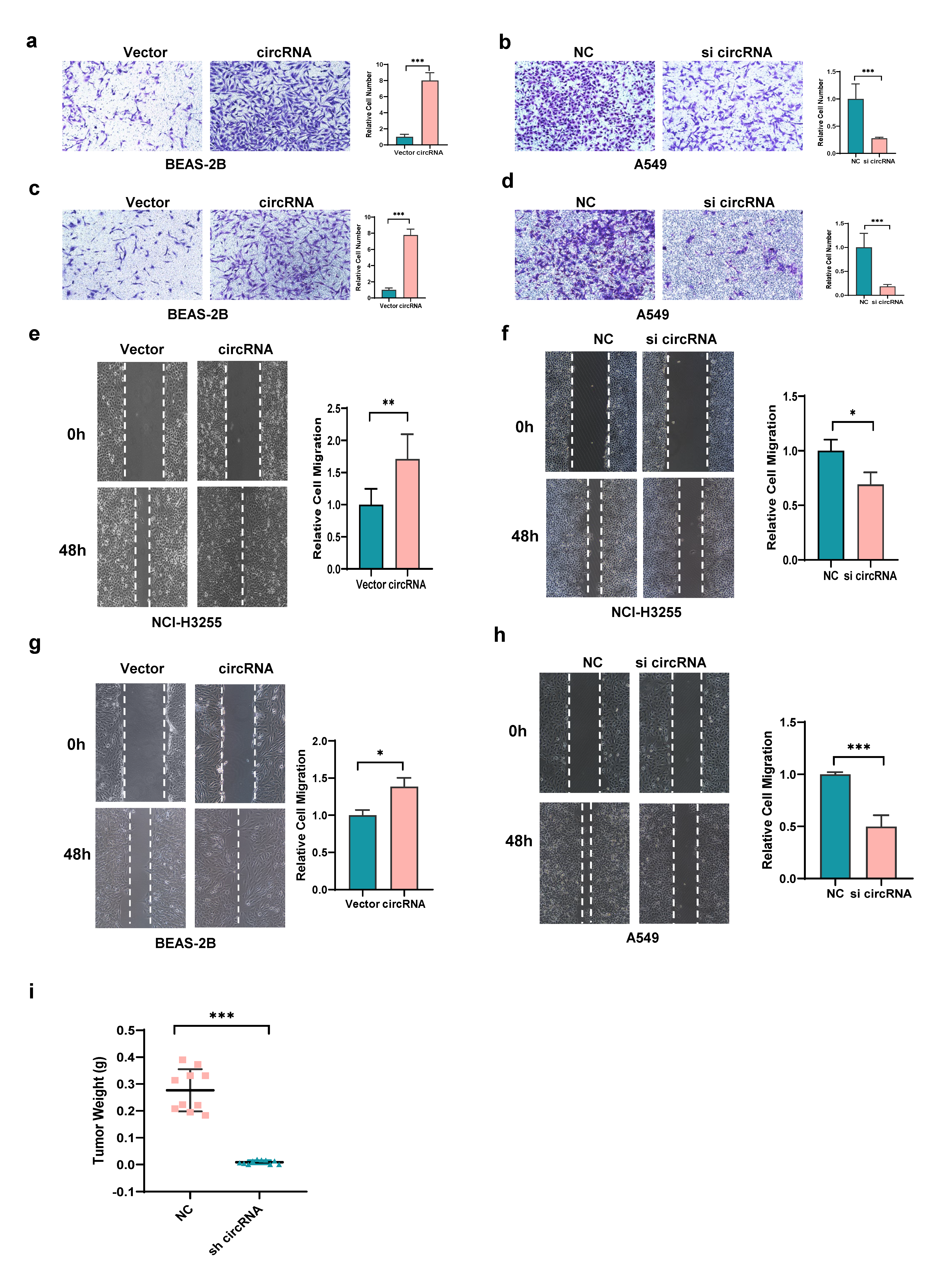

Supplement: Supplementary file 4 — Additional file 4: Figure S3. CircHERC1 promotes NSCLC cell migration and invasion (a-i) [file 12943_2023_1888_MOESM4_ESM.png]

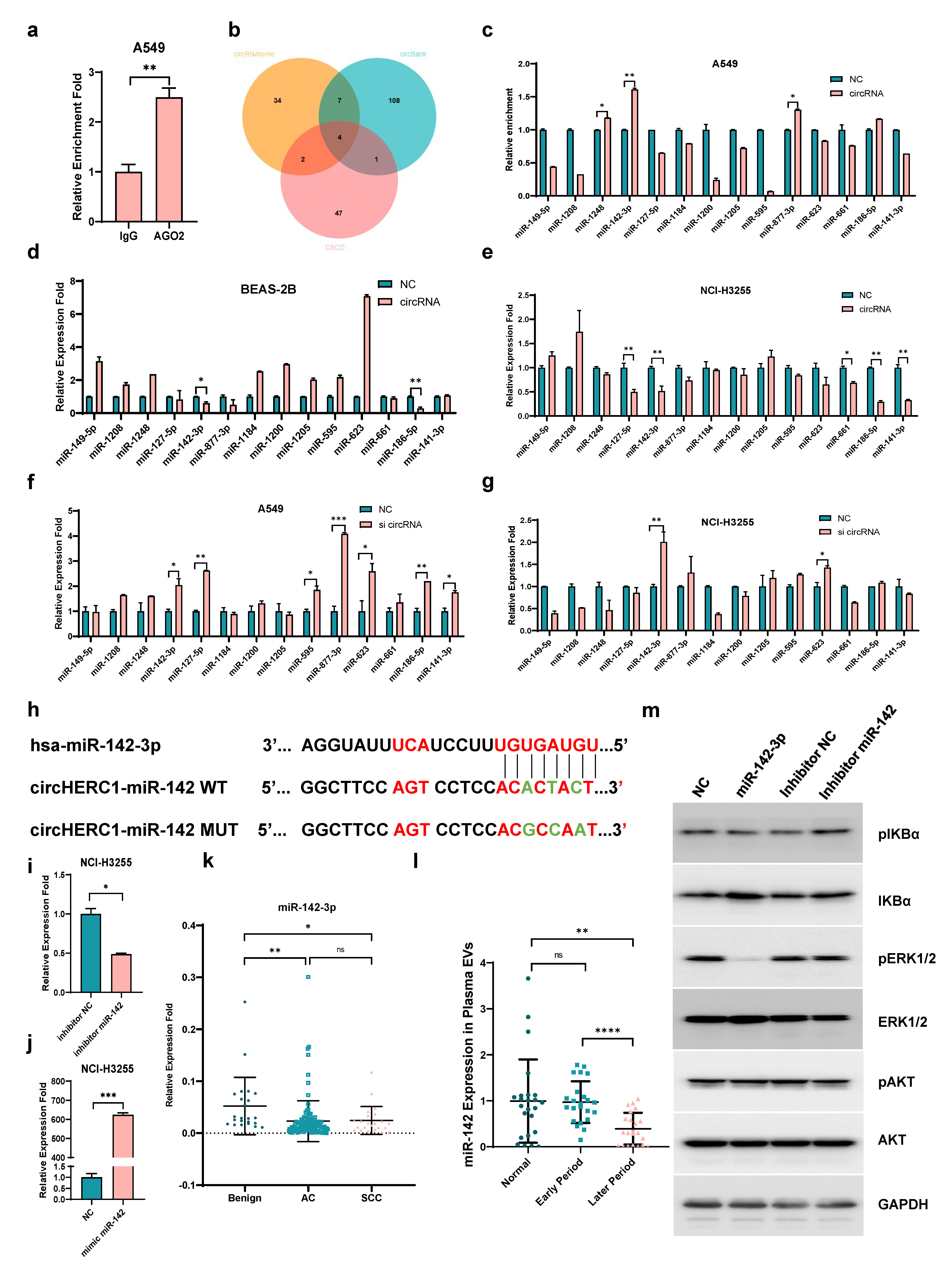

Supplement: Supplementary file 5 — Additional file 5: Figure S4. CircHERC1directly binds miR-142 and regulates miR-142 expression in NSCLC cells(a-m) [file 12943_2023_1888_MOESM5_ESM.png]

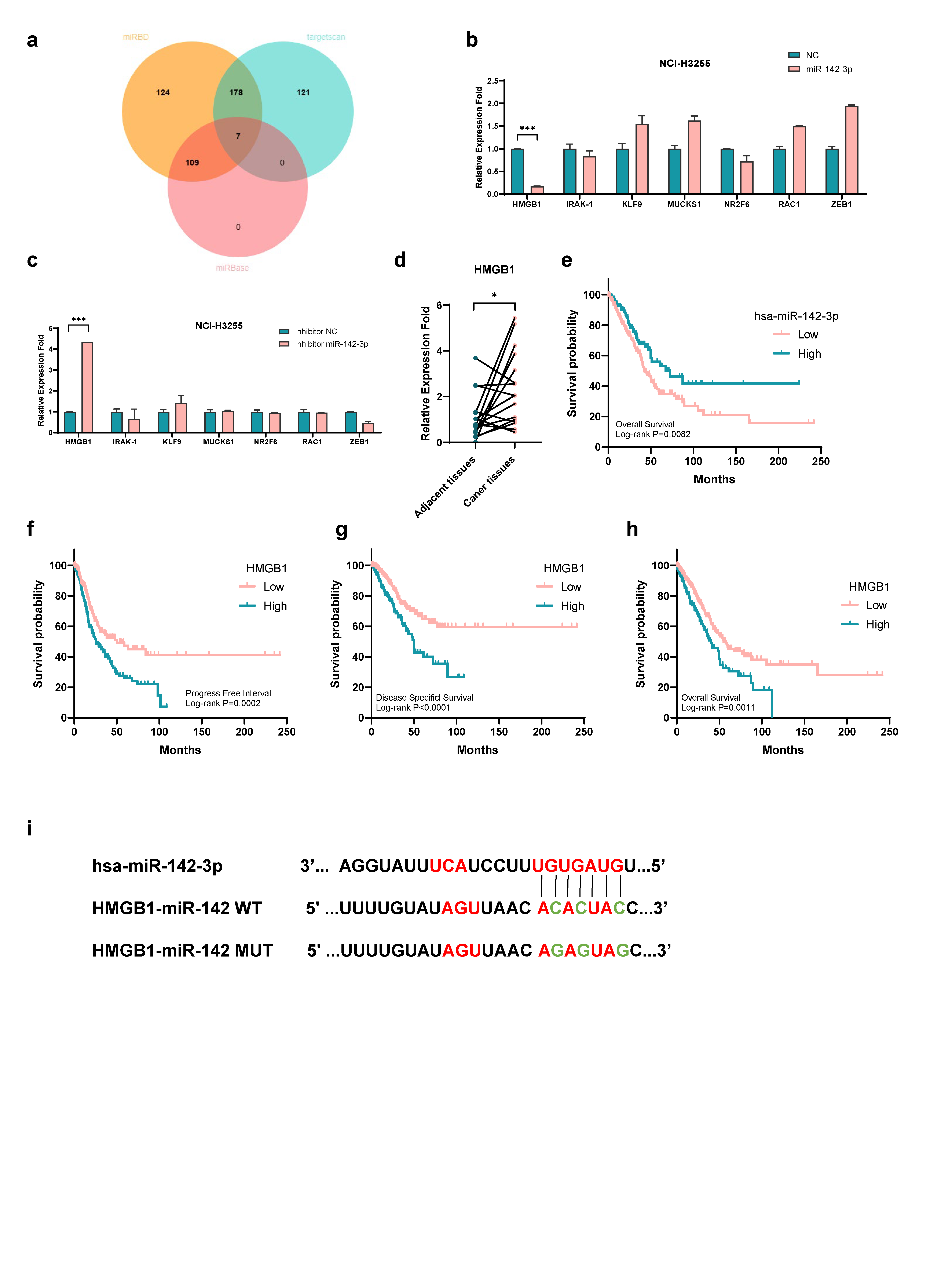

Supplement: Supplementary file 6 — Additional file 6: Figure S5. HMGB1 is a functional target of miR-142-3p(a-i) [file 12943_2023_1888_MOESM6_ESM.png]

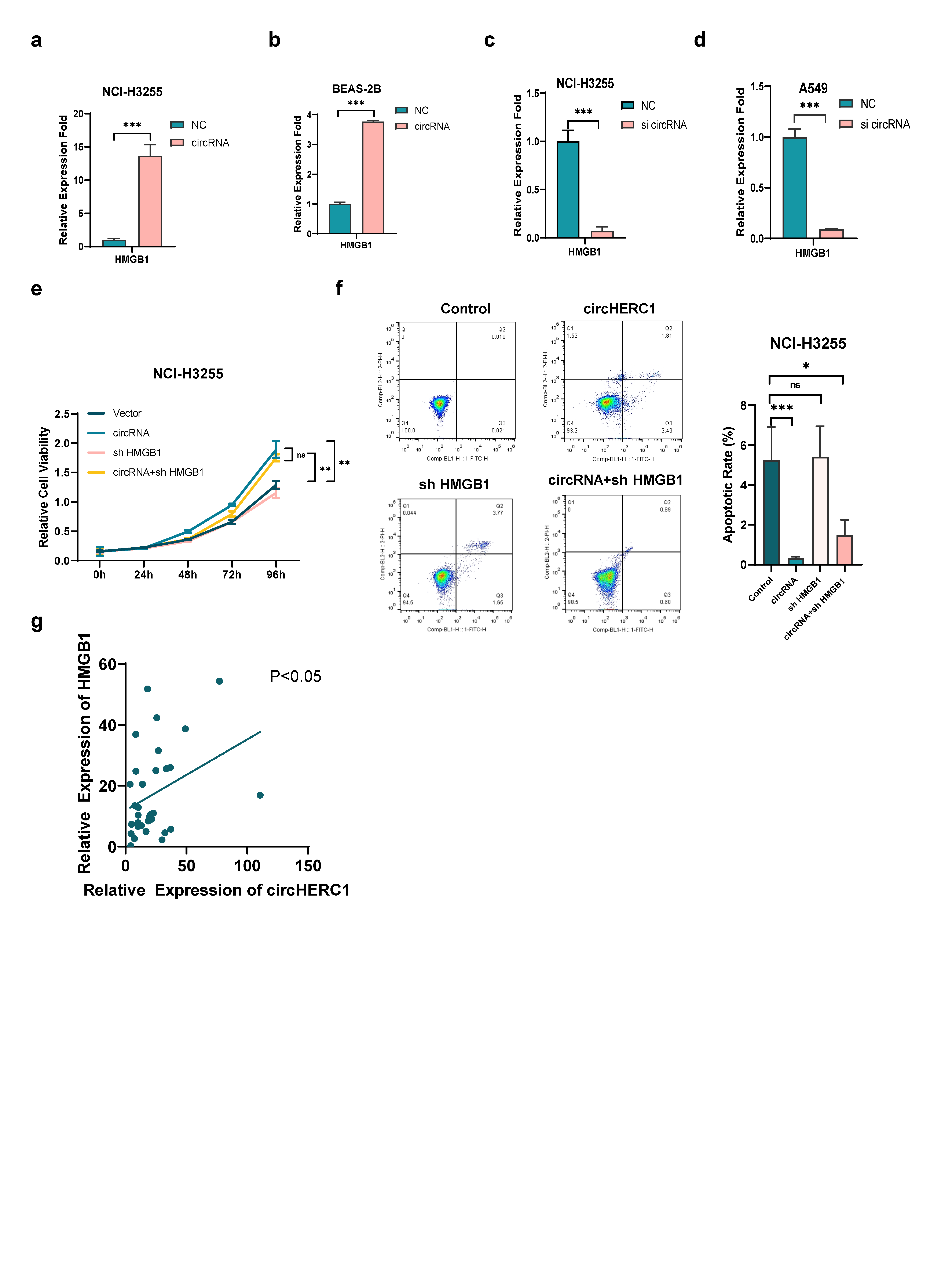

Supplement: Supplementary file 7 — Additional file 7: Figure S6. CircHERC1 upregulates HMGB1 expression by sponging miR-142 (a-g) [file 12943_2023_1888_MOESM7_ESM.png]

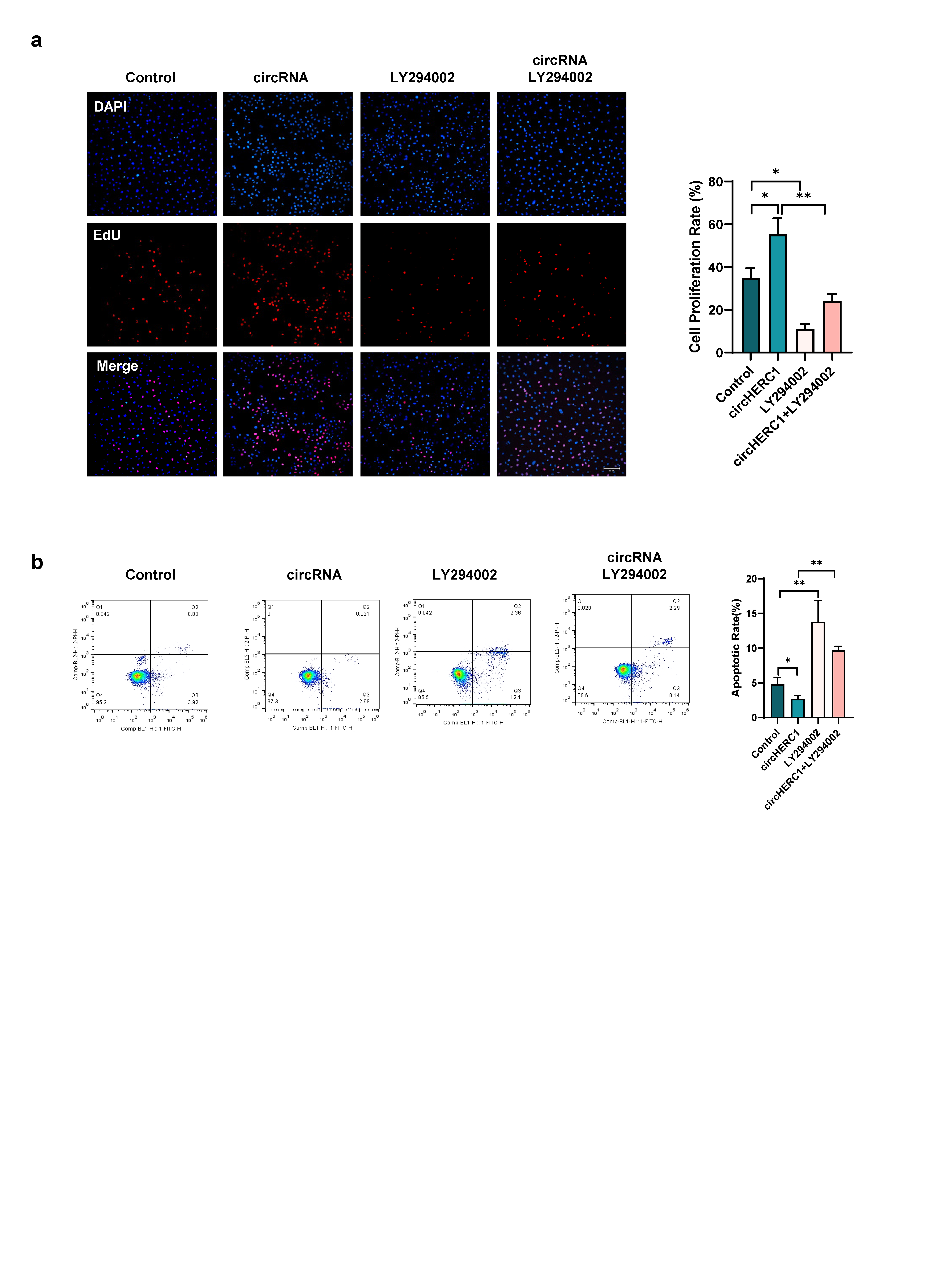

Supplement: Supplementary file 8 — Additional file 8: Figure S7. CircHERC1 elevates cell viability through the PI3K/AKT pathway (a-b) [file 12943_2023_1888_MOESM8_ESM.png]

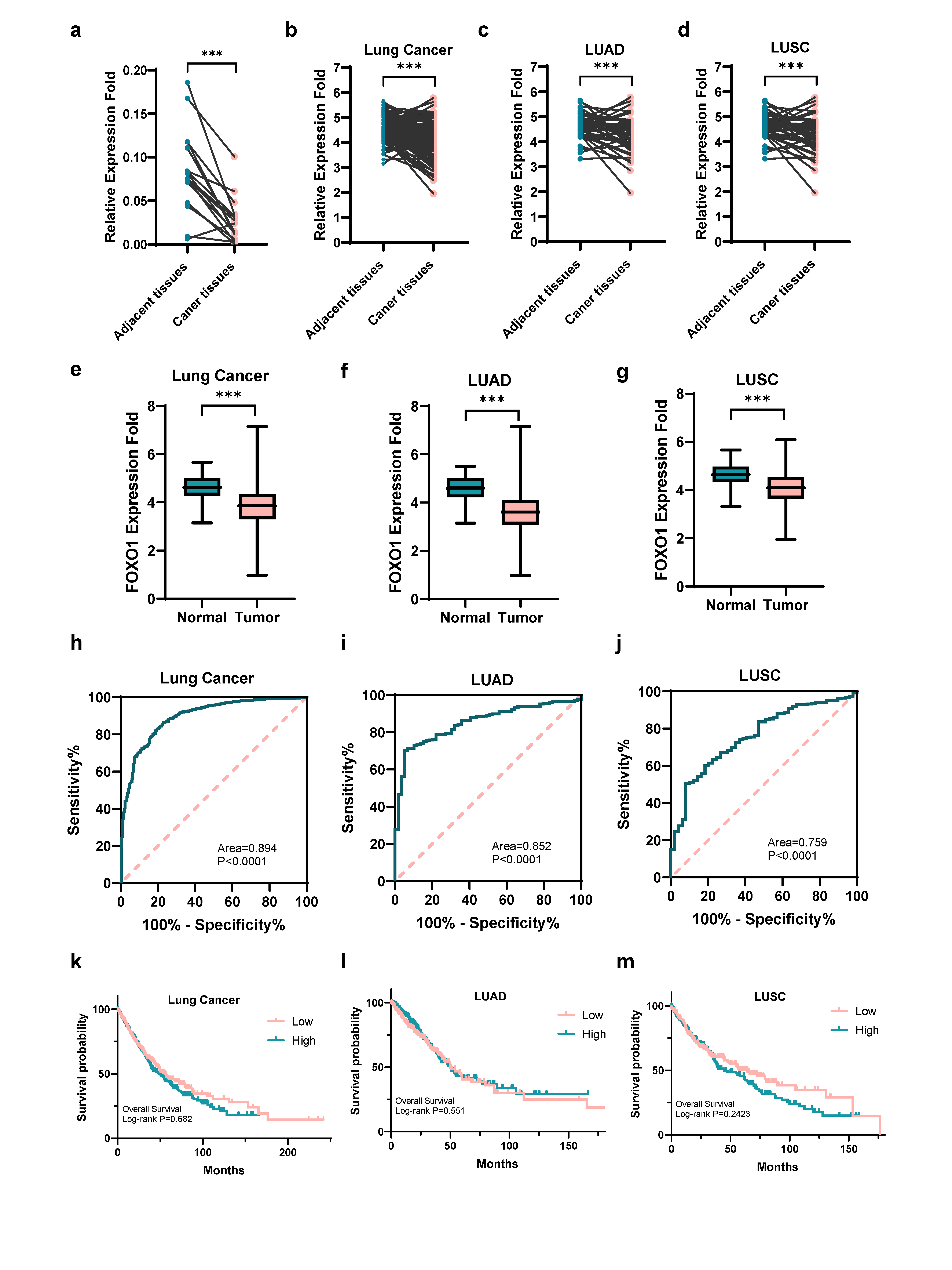

Supplement: Supplementary file 9 — Additional file 9: Figure S8. Down-regulated expression of FOXO1 in lung cancers (a-m) [file 12943_2023_1888_MOESM9_ESM.png]

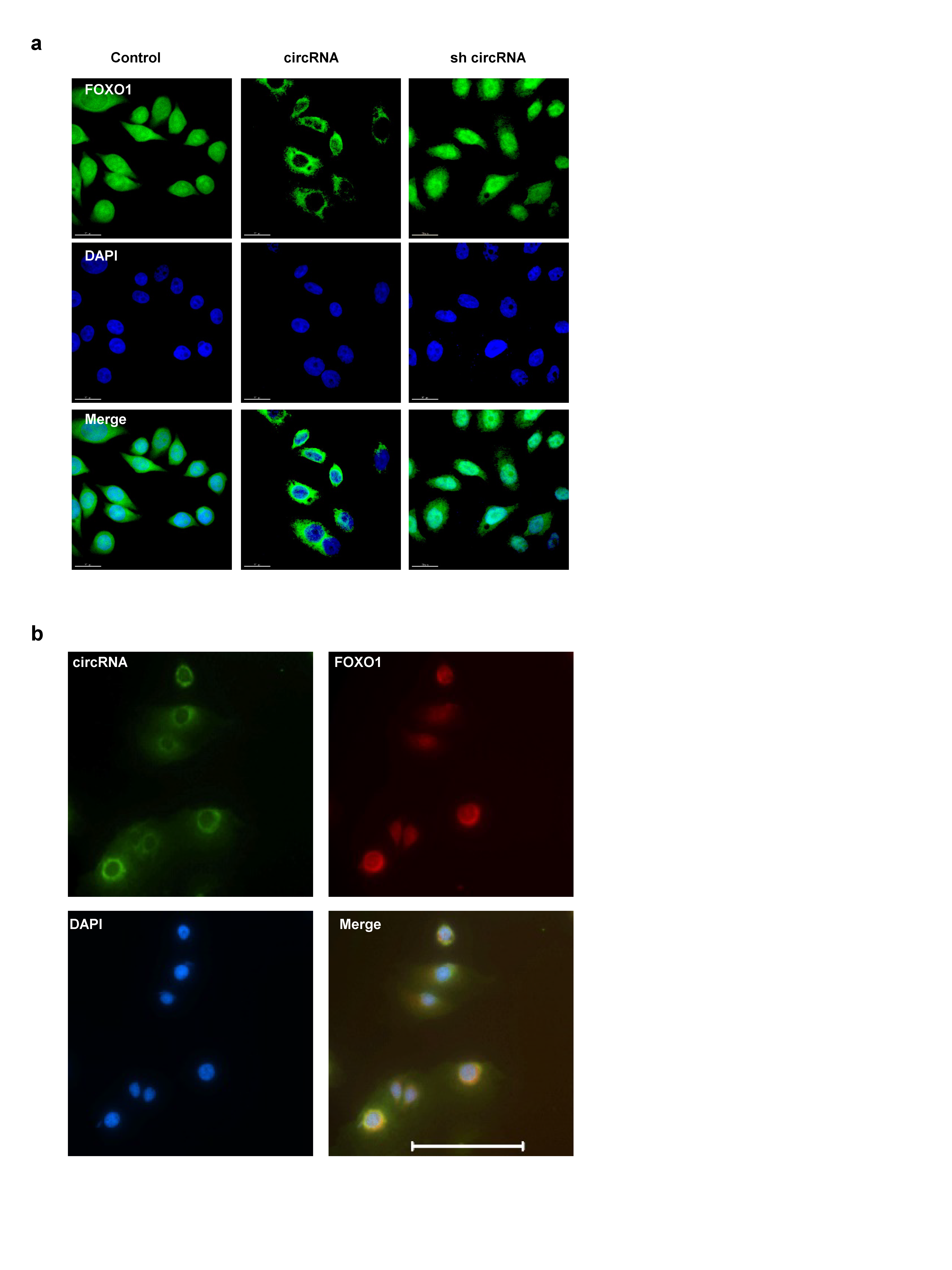

Supplement: Supplementary file 10 — Additional file 10: Figure S9. FOXO1 accumulation in the cytoplasm in circHERC1 overexpressing cells (a-b) [file 12943_2023_1888_MOESM10_ESM.png]

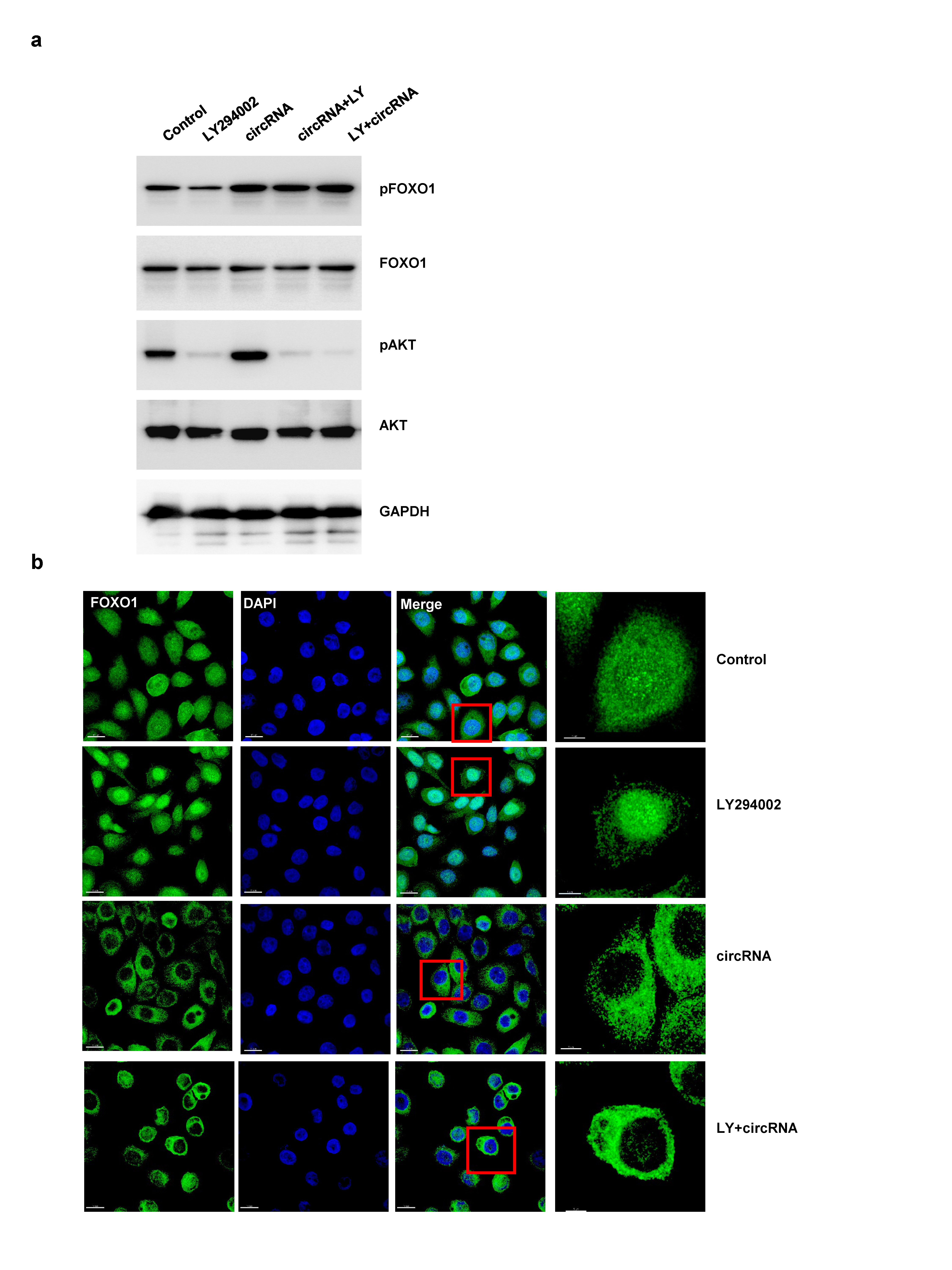

Supplement: Supplementary file 11 — Additional file 11: Figure S10. FOXO1 accumulation in the cytoplasm independent of AKT activation (a-b) [file 12943_2023_1888_MOESM11_ESM.png]

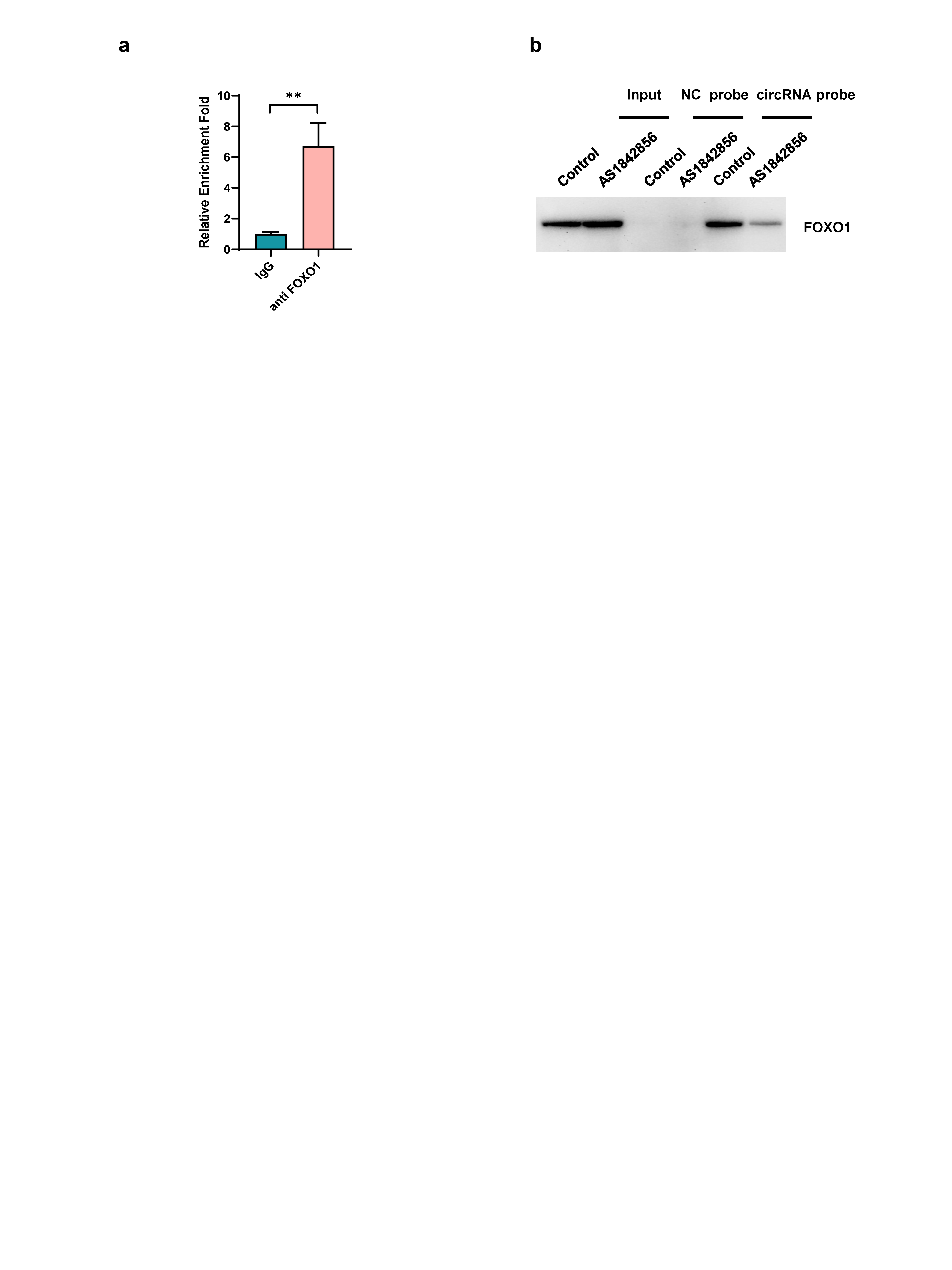

Supplement: Supplementary file 12 — Additional file 12: Figure S11. CircHERC1 interacts with FOXO1 (a-b) [file 12943_2023_1888_MOESM12_ESM.png]

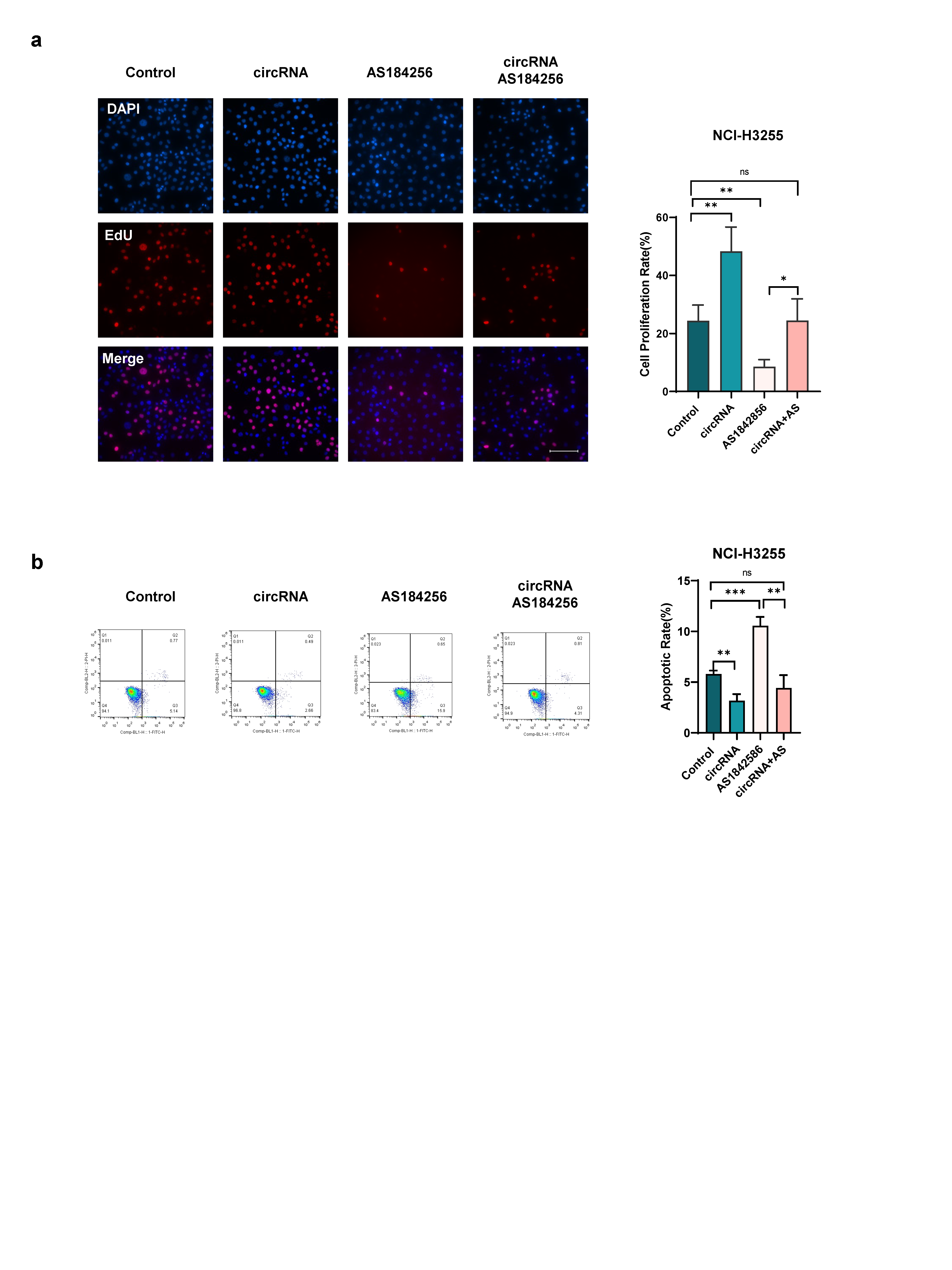

Supplement: Supplementary file 13 — Additional file 13: Figure S12. CircHERC1 elevates cell viability by interacting with FOXO1 (a-b) [file 12943_2023_1888_MOESM13_ESM.png]

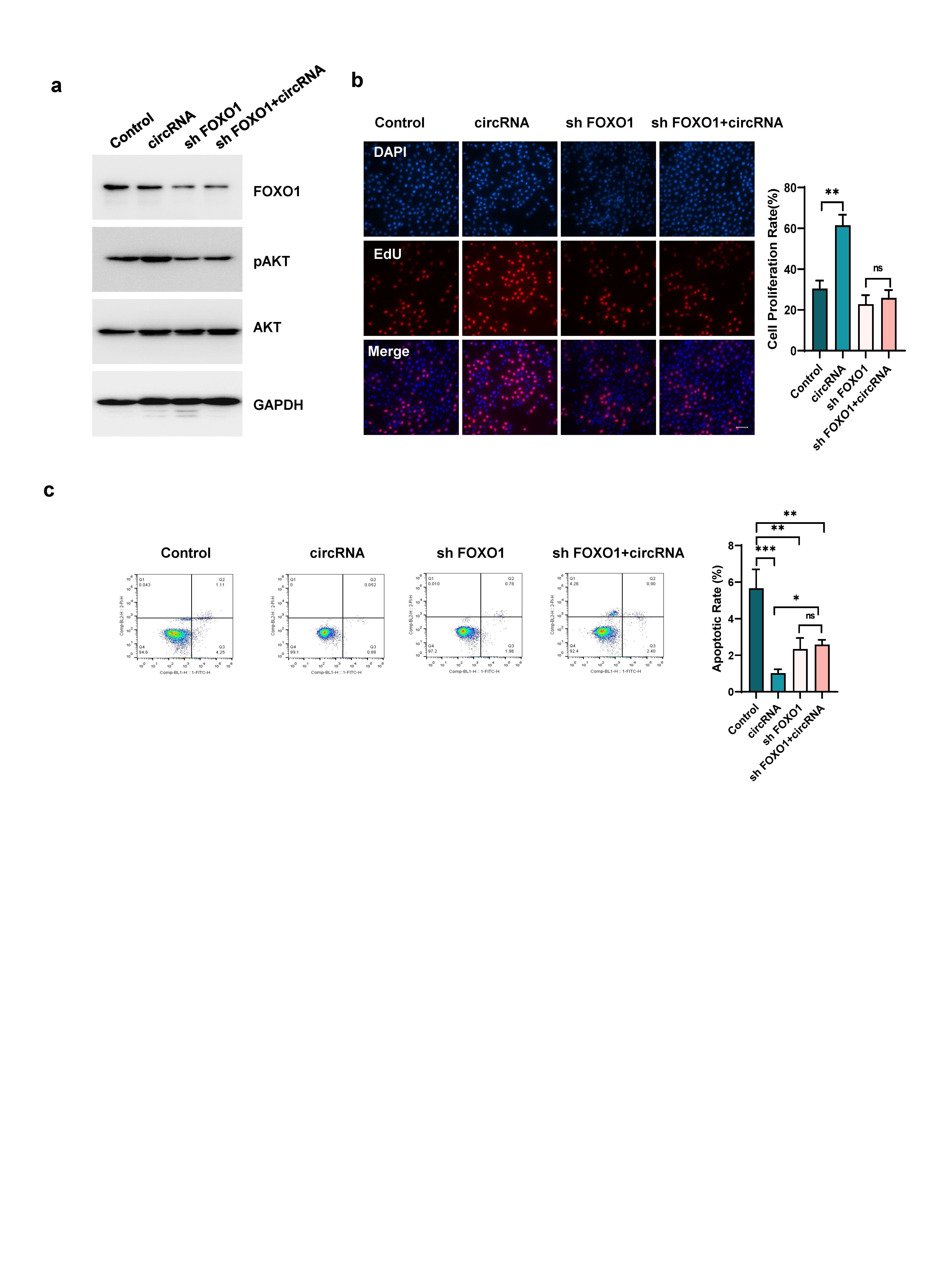

Supplement: Supplementary file 14 — Additional file 14: Figure S13. Inhibition of FOXO1 expression attenuates the influence of circHERC1 on cell proliferation and apoptosis (a-c) [file 12943_2023_1888_MOESM14_ESM.png]

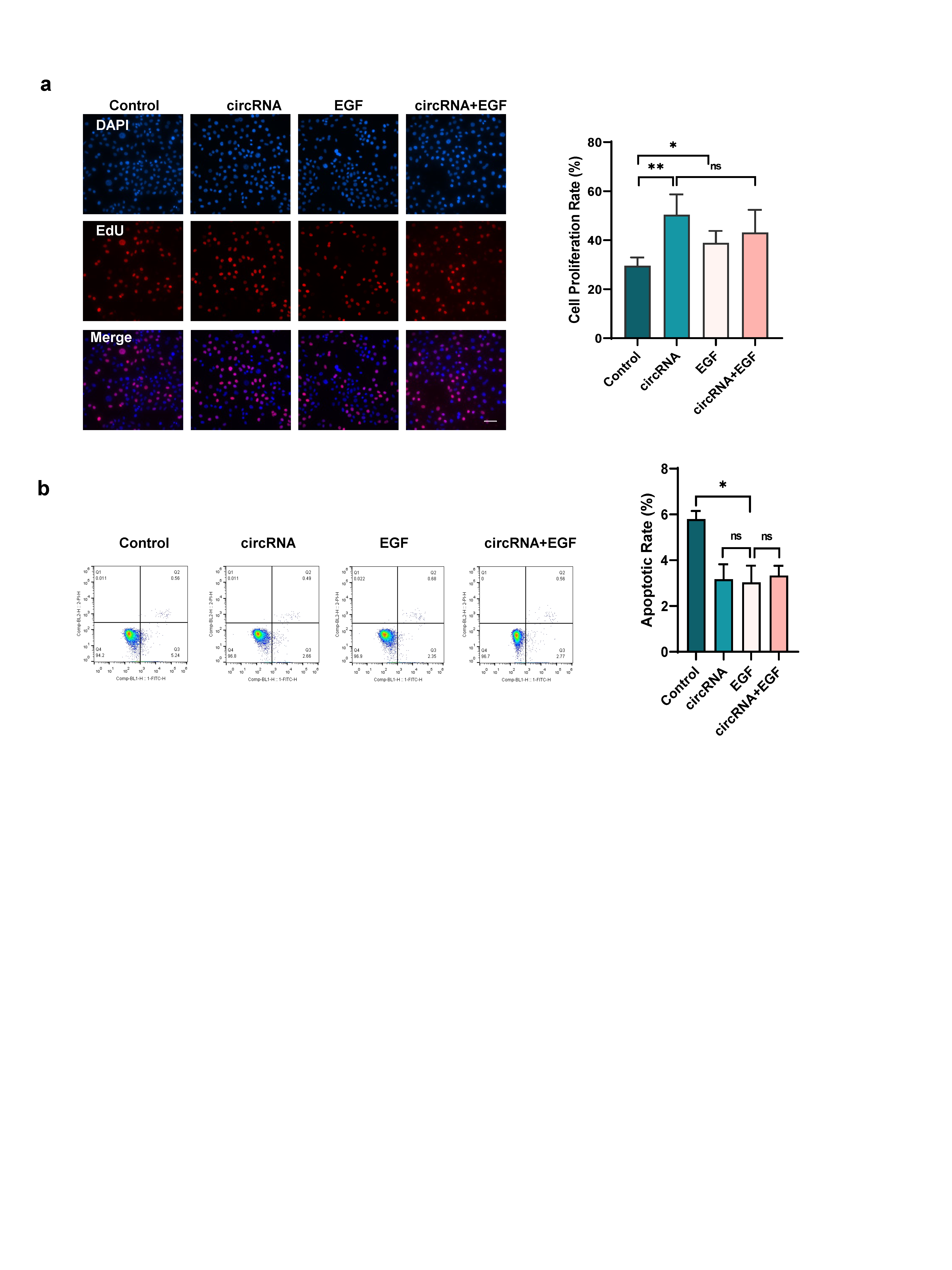

Supplement: Supplementary file 15 — Additional file 15: Figure S14. Feedback AKT activation by FOXO1 leads to loss of reactivity to EGF (a-b) [file 12943_2023_1888_MOESM15_ESM.png]

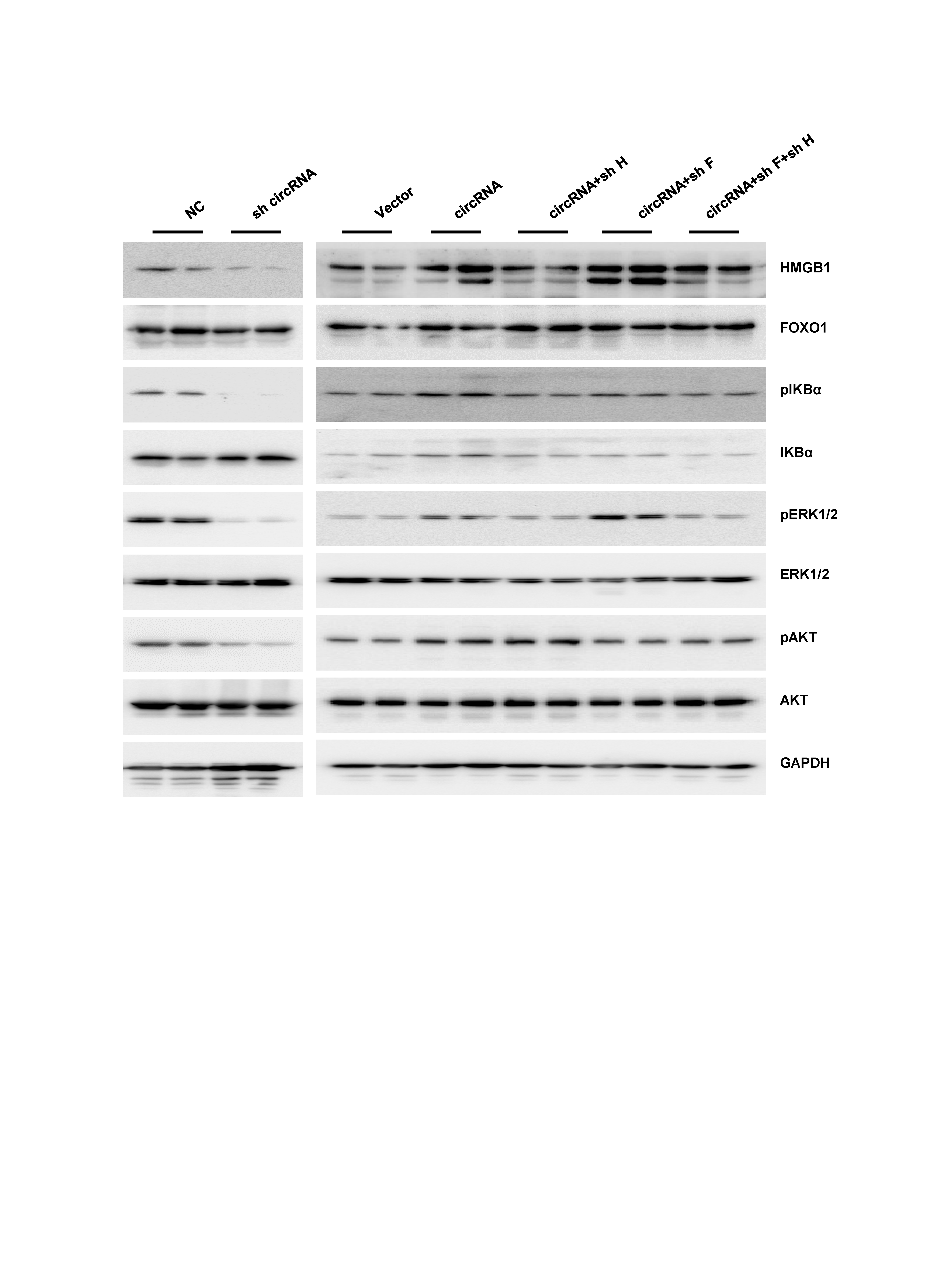

Supplement: Supplementary file 16 — Additional file 16: Figure S15. Western blot analysis of HMGB1, FOXO1, MAPK/ERK, IKBα and PI3K/AKT in xenograft tumors [file 12943_2023_1888_MOESM16_ESM.png]

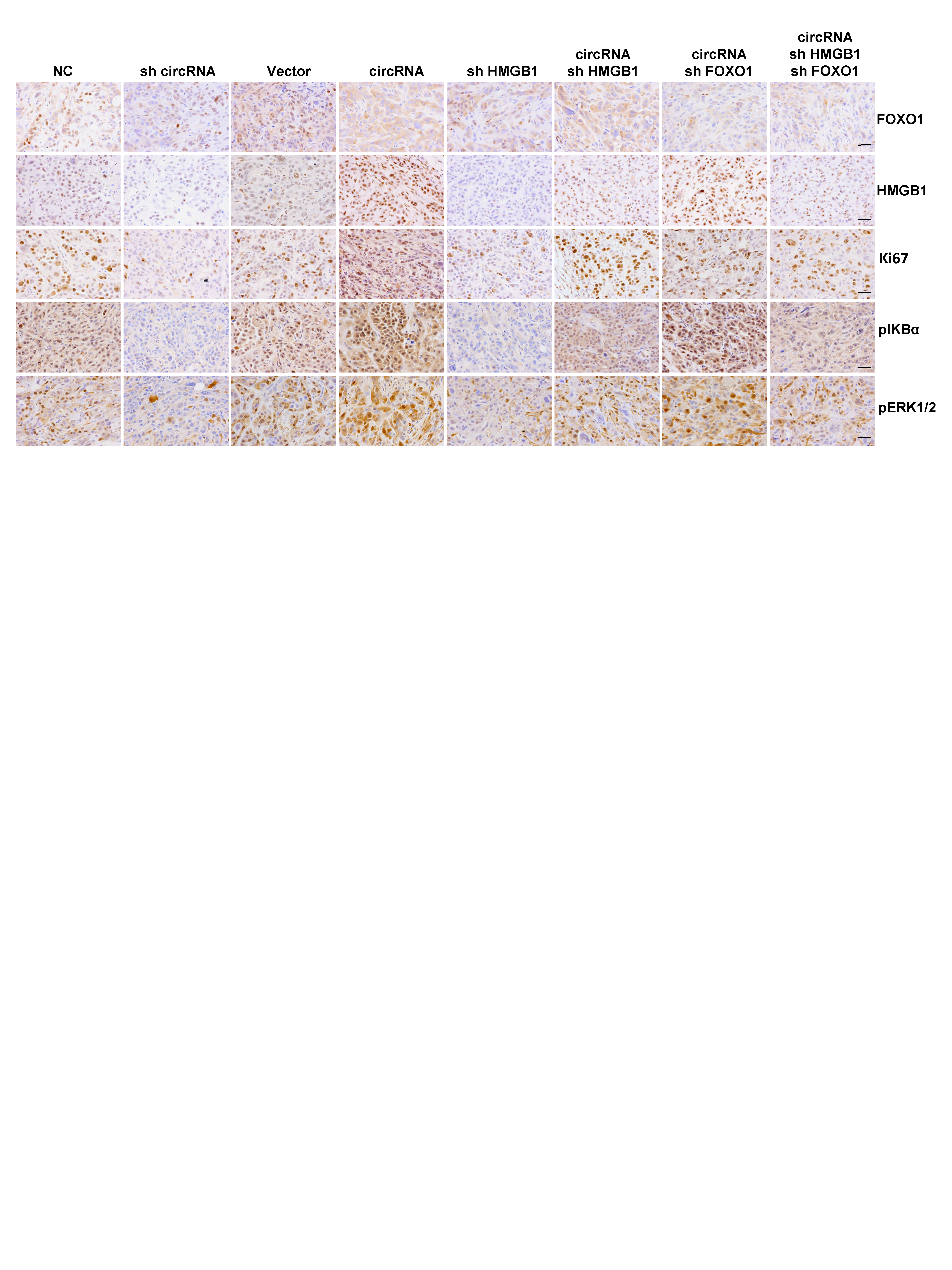

Supplement: Supplementary file 17 — Additional file 17: Figure S16. IHC staining of FOXO1, HMGB1, Ki67, pIKBα and pERK1/2 in xenograft tumors [file 12943_2023_1888_MOESM17_ESM.png]

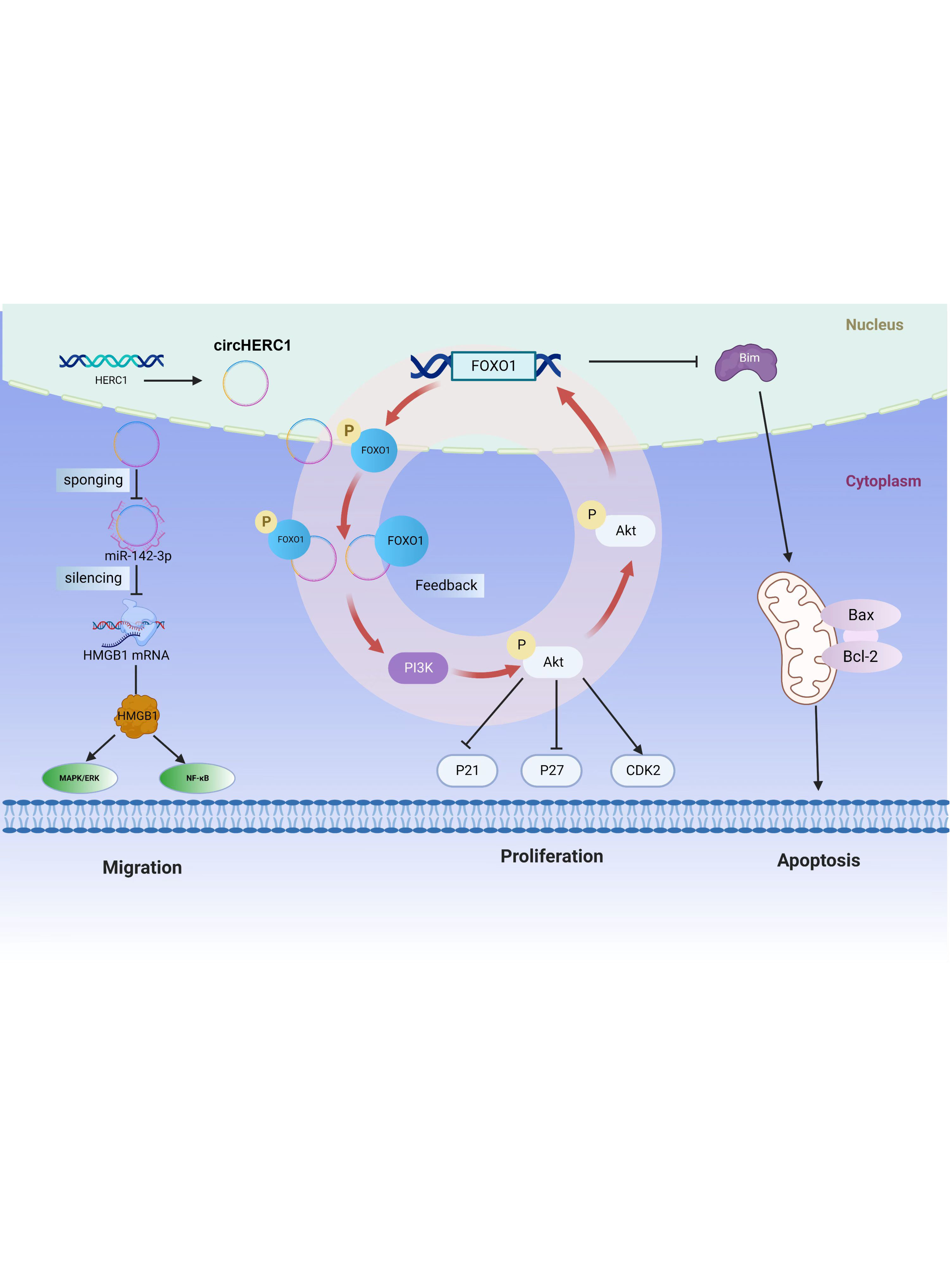

Supplement: Supplementary file 18 — Additional file 18: Figure S17. Schematic diagram illustrating the mechanism [file 12943_2023_1888_MOESM18_ESM.png]
