# Supplementary material for: CircHERC1 promotes non-small cell lung cancer cell progression by sequestering FOXO1 in the cytoplasm and regulating the miR-142-3p-HMGB1 axis
Source: Mol Cancer. 2023 Nov 6;22:179. doi: 10.1186/s12943-023-01888-7 (PMC10626661; doi:10.1186/s12943-023-01888-7)
Supplement: Supplementary file 19 — Supplementary Material 19 [file 12943_2023_1888_MOESM19_ESM.docx]

**Fig. S1** Oncogenic circRNA discovery and characterization of circHERC1 in NSCLC. **a)** qRT‒PCR to assess the stability of circHERC1 and HERC1 under RNase R treatment in A549 cells. ****p* < 0.001. **b, c)** qRT‒PCR analysis of circHERC1, GAPDH, and U6 in the cytoplasm and nucleus in A549 cells. **d)** qRT‒PCR analysis of circHERC1 expression in exosomes extracted from plasma of patients with early- (n=24) and late-stage (n=24) lung cancer and cancer-free individuals (n=24). ***p* < 0.01. **e)** The expression of circHERC1 in different cell lines.

**Fig. S2** CircHERC1 promotes NSCLC cell proliferation. **a, b)** qRT‒PCR analysis of circHERC1 in NCI-H3255 and BEAS-2B cells overexpressing circHERC1. ****p* < 0.001. **c, d)** qRT‒PCR analysis of circHERC1 in NCI-H3255 and A549 cells with circHERC1 knockdown. ****p* < 0.001. **e, f)** Cell proliferation ability of NCI-H3255 and BEAS-2B cells overexpressing circHERC1. ***p* < 0.01, ****p* < 0.001. **g, h)** Cell proliferation ability of NCI-H3255 and BEAS-2B cells with circHERC1 knockdown. **p* < 0.01, ****p* < 0.001. **i, j)** EdU assay to detect cell proliferation of BEAS-2B cells overexpressing circHERC1 and A549 cells with circHERC1 knockdown. **p* < 0.05, ***p* < 0.01. The scale bars are 25 µm. **k, l)** Results of the colony formation assay in NCI-H3255 cells with circHERC1 overexpression or knockdown. **p* < 0.05, ***p* < 0.01.

**Fig. S3** CircHERC1 promotes NSCLC cell migration and invasion. **a, c)** Transwell assay to detect cell migration (a) and invasion (c) of BEAS-2B cells overexpressing circHERC1. ****p* < 0.001. **b, d)** Transwell assay to detect cell migration (b) and invasion (d) of A549 cells with circHERC1 knockdown. ****p* < 0.001. **e, f)** Wound healing assay to detect cell migration ability in NCI-H3255 cells overexpressing or knockdown circHERC1. **p* < 0.05, ***p* < 0.01. **g, h)** Wound healing assay to detect migration ability in BEAS-2B cells overexpressing circHERC1 and A549 cells with circHERC1 knockdown. **p* < 0.05, ****p* < 0.001. **i)** Weights of xenograft tumors in mice injected with NCI-H3255 cells with stable knockdown of circHERC1 and control cells at the endpoint. ****p* < 0.001.

**Fig. S4** CircHERC1 directly binds miR-142 and regulates miR-142 expression in NSCLC cells. **a)** qRT‒PCR analysis of circHERC1 in A549 cells after pulldown of Ago2 by RIP assay. ***p* < 0.01. **b)** Venn diagram showing the mutual candidate target genes of circHERC1 identified by CSCD, circBase and CircInteractome. **c)** qRT‒PCR of the candidate miRNAs expression in circHERC1 overexpressing A549 cell lysates pulled down and enriched with a biotinylated circHERC1-specific probe. **p* < 0.05, ***p* < 0.01. **d, e)** qRT‒PCR analysis of the candidate miRNAs after circHERC1 overexpression in BEAS-2B cells and NCI-H3255 cells. **p* < 0.05, ***p* < 0.01. **f, g)** qRT‒PCR analysis of the candidate miRNAs after circHERC1 knockdown in A549 cells and NCI-H3255 cells. **p* < 0.05, ***p* < 0.01, ****p* < 0.001. **h)** A schematic of wild-type (WT) and mutant (MUT) miR-142-3p binding sites in circHERC1 luciferase reporter vectors. **i, j)** qRT‒PCR analysis of miR-142-3p in NCI-H3255 cells with knockdown or overexpression of miR-142-3p. **p* < 0.05, ****p* < 0.001. **k)** qRT‒PCR was applied to analyze mir-142-3p expression in plasma of LUAD patients (n=111), LUSC patients (n=23) and cancer-free individuals (n=23). **p* < 0.05, ***p* < 0.01. **l)** qRT‒PCR analysis of mir-142-3p expression in exosomes extracted from plasma of patients with early- (n=23) and late-stage (n=22) lung cancer and cancer-free individuals (n=24). ***p* < 0.01, *****p* < 0.0001. **m)** Western blot analysis of the MAPK/ERK, IKBα and PI3K/AKT pathways in NCI-H3255 cells with altered expression of miR-142-3p.

**Fig. S5** HMGB1 is a functional target of miR-142-3p. **a)** Venn diagram showing the candidate target genes of miR-142-3p identified by miRDB, TargetScan and DIANA. **b, c)** qRT‒PCR analysis of candidate mRNA expression in NCI-H3255 cells with overexpression or knockdown of miR-142-3p. ****p* < 0.001. **d)** qRT‒PCR analysis of HMGB1 in tumor tissues and peritumoral tissues (n=16). **p* < 0.05. **e)** Prognostic analysis of LUAD with low (n=374) and high (n=147) miR-142-3p expression from the TCGA database. **f, g, h)** Prognostic analysis of LUAD with low and high HMGB1 expression from the TCGA database (n=535). **i)** Diagram of the 3′UTR of HMGB1 with putative WT and MUT miR-142-3p binding sites.

**Fig. S6** CircHERC1 upregulates HMGB1 expression by sponging miR-142. **a, b)** qRT‒PCR analysis of HMGB1 expression in NCI-H3255 and BEAS-2B cells overexpressing circHERC1. ****p* < 0.001. **c, d)** qRT‒PCR analysis of HMGB1 expression in NCI-H3255 and A549 cells with circHERC1 knockdown. ****p* < 0.001. **e)** Cell proliferation ability of NCI-H3255 cells after alteration of the expression of circHERC1 and HMGB1. ***p* < 0.01. **f)** Flow cytometry to detect cell apoptosis of NCI-H3255 cells after alteration of the expression of circHERC1 and HMGB1. **p* < 0.05, ****p* < 0.001. **g)** qRT‒PCR analysis to assess the relationships among the expression levels of circHERC1 and HMGB1 (n=32).

**Fig. S7** CircHERC1 elevates cell viability through PI3K/AKT pathway. **a)** EdU assay to detect the proliferation of NCI-H3255 cells stably overexpressing circHERC1 treated with or without LY2940002. **p* < 0.05, ***p* < 0.01. The scale bar is 50 µm. **b)** Flow cytometry to detect apoptosis of NCI-H3255 cells stably overexpressing circHERC1 treated with or without LY2940002. **p* < 0.05, ***p* < 0.01.

**Fig. S8** Down-regulated expression of FOXO1 in lung cancers. **a)** qRT‒PCR analysis of FOXO1 in tumor tissues and peritumoral tissues (n=16). ****p* < 0.001. **b, c, d)** The expression of FOXO1 in paired tumor tissues and peritumoral tissues of lung cancer (n=107), LUAD (n=49) and LUSC (n=49) patients from the TCGA database. ****p* < 0.001. **e, f, g)** The expression of FOXO1 in tumor tissues and normal tissues of lung cancer (Normal, n=108, Tumor, n=1041), LUAD (Normal, n=59, Tumor, n=539) and LUSC (Normal, n=49, Tumor, n=502) patients from the TCGA database. ****p* < 0.001. **h, i, j)** ROC curve analysis of FOXO1 expression in lung cancer (Normal, n=397, Tumor, n=1013), LUAD (Normal, n=59, Tumor, n=539) and LUSC (Normal, n=49, Tumor, n=502) patients from the TCGA database. **h, i, j)** Prognostic analysis of lung cancer (High, n=521, Low, n=520), LUAD (High, n=288, Low, n=251) and LUSC (High, n=251, Low, n=251) with low and high FOXO1 expression from the TCGA database.

**Fig. S9** FOXO1 accumulation in the cytoplasm in circHERC1 overexpressing cells. **a)** IF assays to detect FOXO1 subcellular localization in NCI-H3255 cells transfected with the control vector, the circHERC1 overexpression vector and the circHERC1 knockdown vector. The scale bars are 20 µm. **b)** FISH to detect the subcellular location change of FOXO1 in A549 cells transfected with the circHERC1 overexpression vector. The scale bar is 25 µm.

**Fig. S10** FOXO1 accumulation in the cytoplasm independent of AKT activation. **a)** Western blot analysis of FOXO1 and AKT expression in NCI-H3255 cells after alteration of the expression of circHERC1 and treatment with LY294002. CircRNA+LY represents circHERC1 overexpressing cells treated with LY294002. LY+circRNA represents cells pretreated with LY294002, then overexpressed circHERC1. **b)** IF assays to detect FOXO1 subcellular localization in A549 cells after alteration of the expression of circHERC1 and treatment with LY294002. The scale bars are 15 µm.

**Fig. S11** CircHERC1 interacts with FOXO1. **a)** RIP assays to evaluate the interaction between circHERC1 and FOXO1 using a FOXO1 antibody. ***p* < 0.01. **b)** RNA pulldown assays to evaluate the interaction between circHERC1 and FOXO1 using biotinylated probes against circHERC1.

**Fig. S12** CircHERC1 elevates cell viability by interacting with FOXO1. **a)** EdU assay to detect the proliferation of NCI-H3255 cells stably overexpressing circHERC1 treated with or without AS184256. **p* < 0.05, ***p* < 0.01. The scale bar is 25 µm. **b)** Flow cytometry to detect apoptosis of NCI-H3255 cells stably overexpressing circHERC1. treated with or without AS184256. **p* < 0.05, ***p* < 0.01.

**Fig. S13** Inhibition of FOXO1 expression attenuates the influence of circHERC1 on cell proliferation and apoptosis. **a)** Western blot analysis of FOXO1 and AKT expression in NCI-H3255 cells after alteration of the expression of circHERC1 or FOXO1. **b)** EdU assay to detect the proliferation of NCI-H3255 cells after alteration of the expression of circHERC1 or FOXO1. ***p* < 0.01. The scale bar is 25 µm. **c)** Flow cytometry to detect apoptosis of NCI-H3255 cells after alteration of the expression of circHERC1 or FOXO1. **p* < 0.05, ***p* < 0.01, ****p* < 0.001.

**Fig. S14** Feedback AKT activation by FOXO1 leads to loss of reactivity to EGF. **a)** EdU assay to detect the proliferation of NCI-H3255 cells altered expression of circHERC1 and added EGF using. **p* < 0.05, ***p* < 0.01. Sacle bar is 25 µm. **b)** Flow cytometry to detect the cell apoptosis of NCI-H3255 cells altered expression of circHERC1 and added EGF. **p* < 0.05.

**Fig. S15** Western blot analysis of HMGB1, FOXO1, MAPK/ERK, IKBα and PI3K/AKT in xenograft tumors.

**Fig. S16** IHC staining of FOXO1, HMGB1, Ki67, pIKBα and pERK1/2 in xenograft tumors. The scale bars are 25 µm.

**Fig. S17** Schematic diagram illustrating the mechanism by which circHERC1 promotes NSCLC proliferation and metastasis via the miR-142-3p-HMGB1 axis and interaction with FOXO1.
